# USP35 Acts as a Deubiquitinating Enzyme for ID3 to Promote Immune Escape in Colorectal Cancer

**DOI:** 10.1002/advs.202516588

**Published:** 2026-01-04

**Authors:** Wenxin Chen, Ling Wang, Hongmei Fan, Lisheng Li, Lingyu Zhang, Ruoxin Li, Fang Liu, Fuli Wen, Yunbin Ye, Chuanzhong Huang

**Affiliations:** ^1^ Laboratory of Immuno‐Oncology, Clinical Oncology School of Fujian Medical University Fujian Cancer Hospital Fuzhou P. R. China; ^2^ School of Basic Medical Sciences Fujian Medical University Fuzhou P. R. China; ^3^ Shengli Clinical Medical College of Fujian Medical University, Fujian Provincial Hospital Fuzhou University Affiliated Provincial Hospital Fuzhou P. R. China; ^4^ NHC Key Laboratory of Cancer Metabolism (Fujian Cancer Hospital), Fujian Key Laboratory of Translational Cancer Medicine Fuzhou P. R. China

**Keywords:** deubiquitination, ID3, immune evasion, IU1, USP35

## Abstract

Our prior investigation has demonstrated that ID3 modulates PD‐L1, thereby affecting immune evasion in colorectal cancer (CRC). Nonetheless, the regulatory mechanisms of ID3, particularly those involving its ubiquitination and degradation, remain inadequately understood. In this study, we identify USP35 as a deubiquitinating enzyme for ID3, which stabilizes ID3 by targeting the N‐terminal lysine residues K2 and K30, thus inhibiting its ubiquitination and degradation. This stabilization enhances ID3's transcriptional activity, resulting in elevated PD‐L1 expression in CRC and facilitating immune escape. Furthermore, we discover an effective inhibitor of USP35 enzyme activity, IU1, which directly suppresses ID3 expression by promoting its ubiquitination and subsequently reducing PD‐L1 expression. IU1 not only impedes tumor cell proliferation, but also augments the efficacy of PD‐L1 monoclonal antibody therapy by enhancing anti‐tumor immune responses. These findings elucidate a novel mechanism by which the USP35‐ID3‐PD‐L1 axis contributes to immune evasion in CRC and propose a potential therapeutic strategy to improve the efficacy of immunotherapy in CRC patients.

## Graphical abstract

USP35 stabilizes ID3 expression by deubiquitinating the K2/K30 site, thereby upregulating PD‐L1 and promoting immune escape in colorectal cancer. IU1, an inhibitor of USP35 enzyme activity, has been shown to inhibit USP35, thereby accelerating ID3 degradation, enhancing CD8^+^ T cell killing, and reversing the immunosuppressive microenvironment.

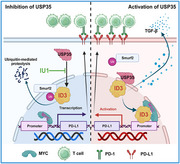

## 1 Introduction

Colorectal cancer (CRC) is one of the most prevalent gastrointestinal malignancies that is reported worldwide [1]. The high incidence of this condition underscores the pressing need for a comprehensive understanding of its etiological characteristics and molecular mechanisms [2]. A mounting body of evidence indicates that immune system dysfunction plays a pivotal role in the initiation, progression, and metastasis of CRC [3]. This dysfunction not only fosters a microenvironment conducive to tumor cell proliferation and invasion but also accelerates disease progression by suppressing the host's antitumor immune response [4]. This immune regulatory abnormality is intertwined with pathological processes such as genetic mutations, epigenetic alterations, and dysregulation of immune checkpoint molecules [5, 6]. Collectively, these factors form the complex, multidimensional pathogenesis network of CRC.

The ubiquitin‐proteasome system (UPS) is a crucial post‐translational modification mechanism, accounting for approximately 80%‐90% of protein degradation processes in eukaryotic cells [7, 8]. This system facilitates the targeting of ubiquitin molecules to proteins through a cascade reaction involving the E1, E2, and E3 enzymes, and dynamically removes ubiquitin modifications via deubiquitinating enzymes (DUB), thereby forming a finely balanced regulatory network that governs various biological functions such as protein degradation, activation, subcellular localization, and protein‐protein interactions [9]. The UPS plays a vital role in maintaining normal cell function and ensuring human health. In the domain of tumor immunology [10], ubiquitination not only regulates key immune response processes such as dendritic cell maturation [11], antigen presentation efficiency [12], and interactions with T cells [13], but also significantly mediates PD‐L1 expression regulation and the NF‐κB signaling pathway activity in tumor cells [14, 15].

Inhibitors targeting the ubiquitination pathway have shown substantial preclinical treatment potential when combined with immunotherapy. For instance, inhibitors of the E3 ubiquitin ligase IAP can enhance tumor cells' sensitivity to T cell‐mediated killing and augment the antitumor activity of CAR‐T cells [16, 17]. MDM2 inhibitors inhibit the ubiquitination and degradation of p53 and when used in combination with PD‐1 antibodies, have demonstrated synergistic antitumor effects in cervical cancer treatment [18]. These findings clearly indicate that ubiquitination modifications hold promise for breakthrough directions in tumor immunotherapy.

Our previous research has shown that tumor cell‐expressed ID3 promotes the binding of MYC to the PD‐L1 promoter by restructuring the 4D structure of MYC, thereby enhancing the transcriptional activity of PD‐L1, increasing PD‐L1 expression, and further inhibiting the infiltration and activation of CD8^+^ T cells, promoting immune escape of CRC cells [19]. ID3, a member of the Inhibitor of DNA binding (ID) family, also referred to as the Inhibitor of differentiation, acts as a crucial regulatory switch for a multitude of genes in immune cells [20]. It is integral to the differentiation, development, and apoptosis of immune cells and plays a role in regulating the antitumor functions of CAR‐T cells and macrophages [21, 22]. Importantly, ID3 is often overexpressed in tumor tissues and is closely linked to tumor aggressiveness and a poor clinical prognosis [23]. It also contributes to drug resistance in melanoma to vemurafenib, bladder cancer to erlotinib, and esophageal cancer to cisplatin by influencing tumor cell stemness [24, 25, 26]. Recent advancements in ID3‐related research are increasingly highlighting its pivotal role in tumorigenesis and progression.

Despite extensive research, the regulatory mechanisms governing ID3 remain inadequately understood, with existing studies predominantly concentrating on the identification of indirect regulatory pathways. These pathways encompass epigenetic modifications, such as histone acetylation and methylation that can influence ID3 gene transcription [27], cytokines like IL‐2 and IL‐7 that regulate its expression through signaling pathways [28], and the transcription factor Tcf1 that indirectly affects ID3 expression by modulating the Bcl6‐Blimp1 axis [29]. However, investigations into the direct regulatory mechanisms of ID3 are limited, particularly concerning post‐translational modifications. Currently, phosphorylation is the only reported modification [30, 31], and there is a notable lack of systematic studies on other types of critical modification, such as ubiquitination. Most importantly, the absence of specific inhibitors that directly target ID3 significantly constrains research on precision therapies targeting ID3. Addressing these bottlenecks is of paramount importance.

In this study we discovered that the ID3 protein is mainly degraded through the ubiquitin‐proteasome pathway and successfully screened out USP35 as one of its deubiquitinating enzymes. By targeting the K2 and K30 lysine residues located at the N‐terminal of the ID3 protein, the process of ubiquitination degradation was significantly inhibited, thereby enhancing the stability of the ID3 protein. And then, it is imperative to enhance the expression of PD‐L1 through the utilization of transcriptional regulatory mechanisms, thereby facilitating immune evasion in CRC. Furthermore, the ID3 inhibitor IU1 has been identified that has been shown to not only effectively inhibit the proliferation of CRC cells, but also significantly enhance the sensitivity of CRC to immunotherapy. This provides important theoretical support and practical guidance for improving the treatment response rate and quality of life of CRC patients.

## 2 Results

### 2.1 USP35 Acts as a Deubiquitinating Enzyme to Regulate the Stability of ID3

**FIGURE 1:**
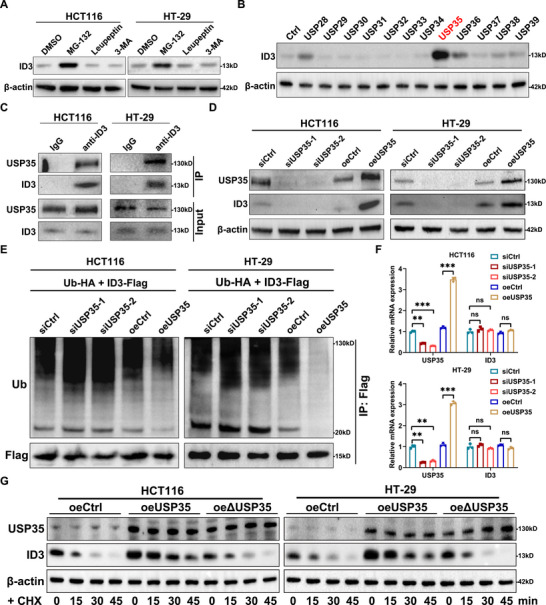
FIGURE 1 USP35 regulates ID3 protein stability as a deubiquitinating enzyme. (A) Western blot analysis illustrates the impact of proteasome inhibitor MG‐132, lysosomal inhibitor Leupeptin, and autophagy inhibitor 3‐MA on ID3 protein level in CRC cells. (B) Application of a DUB overexpression plasmid library, followed by Western blot screening, identifies USP35 as a potential deubiquitinase of ID3. (C) Endogenous Co‐IP analysis reveals an interaction between endogenous USP35 and ID3 in CRC cells. (D, E) Western blot analysis illustrates the impact of USP35 expression on ID3 (D) and its ubiquitination levels (E) in human CRC cells. (F) RT‐qPCR analysis illustrates the impact of USP35 on ID3 mRNA levels in human CRC cells. (G) The half‐life of ID3 protein after overexpression wild‐type and catalytically inactive USP35 in CRC cells. ΔUSP35, catalytically inactive USP35; 3‐MA, 3‐Methyladenine; Ub, ubiquitination; CHX, Cycloheximide. Data are presented as means ± SD. ** *p* < 0.01, *** *p* < 0.001, based on Student's *t* test.

To comprehensively investigated the regulatory mechanism of the ID3 protein, we initially treated CRC cells with the proteasome inhibitor MG‐132, the lysosomal inhibitor Leupeptin, and the autophagy inhibitor 3‐MA to systematically examine the degradation pathway of the ID3 protein. The experimental results indicated that only MG‐132 significantly elevates the level of ID3 protein (Figure 1A), a phenomenon observed not only in CRC cells but also corroborated in various other cancer cell types (Figure S1A). Notably, MG‐132 treatment did not affect the expression level of ID3 mRNA (Figure S1B). Further investigation revealed that the half‐life of the ID3 protein was remarkably short, merely 15 min (Figure S1C), a characteristic consistent with transcription factors that rapidly degrade via the ubiquitin‐proteasome system, suggesting that ID3 may be regulated by the ubiquitin‐proteasome pathway in cancer cells.

Although previous study has suggested that Smurf2 may serve as the E3 ubiquitin ligase for ID3 [32], there have been no reports identifying the deubiquitinating enzyme of ID3. To address this, we constructed an overexpression plasmid library comprising 109 deubiquitinases and screened for those affecting the expression of ID3 protein in 293T cells using Western blot analysis. Through systematic screening, we identified USP35 as significantly up‐regulating the protein level of ID3, indicating it may be one of the deubiquitinating enzymes of ID3 (Figure 1B). To validate this finding we first confirmed the direct interaction between USP35 and ID3 through Co‐IP experiments (Figure 1C). Subsequently, in the CRC cell model the knockdown of USP35 significantly reduced the ID3 protein level and increased its ubiquitination, whereas overexpression of USP35 exhibited the opposite effect, without significantly impacting the expression of ID3 mRNA (Figure 1D–F). Further analysis revealed that overexpression of wild‐type USP35 markedly prolonged the half‐life of endogenous ID3, whereas expression of the catalytically inactive USP35 mutant had no such effect (Figure 1G). Clinical sample analysis also revealed a significant positive correlation between the expression levels of USP35 and ID3 proteins in human CRC tissues (Figure S1D, E). To determine whether this regulatory axis is conserved across malignancies, we examined five cancer cell lines of diverse origin and found that USP35 consistently up‐regulated ID3 expression in each cell line (Figure S1F). These experimental results collectively demonstrate that USP35, as a deubiquitinating enzyme of ID3, plays a significant biological role in CRC cells by regulating the stability of the ID3 protein.

### 2.2 USP35 Promotes CRC Progression by Enhancing Tumor Proliferation and Immune Evasion

**FIGURE 2:**
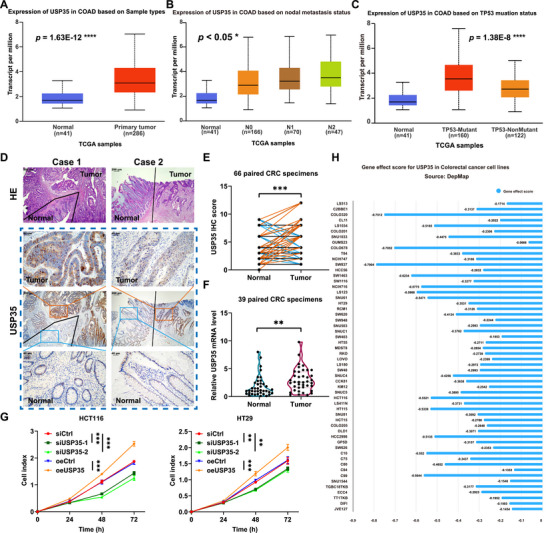
FIGURE 2 USP35 is closely related to the development of CRC. (A) Analysis using the UALCAN platform with TCGA database reveals that USP35 is highly expressed in CRC. (B, C). TCGA database analysis shows that USP35 level is significantly positively correlated with tumor N stage (B) and TP53 mutation status (C). (D, E). Representative immunohistochemistry results (D) and corresponding statistical analysis (E) of USP35 expression in CRC and adjacent non‐tumorous tissues. n = 66. (F). Relative mRNA levels of USP35 in 39 paired CRC tumor tissues compared to adjacent non‐tumorous tissues, as determined by RT‐qPCR. (G). The viability of CRC cells transfected with siUSP35 or pcDNA3.1/USP35 are detected by CCK8 assays. (H). DepMap database analysis shows that the proliferation capacity of all CRC cells is significantly inhibited after USP35 knockdown. si, siRNA; oe, overexpression. Data are presented as means ± SD. * *p* < 0.05, ** *p* < 0.01, *** *p* < 0.001, **** *p* < 0.0001, based on Student's *t* test (A, C, E–G) and Pearson *r* test (B).

An analysis of the TCGA database revealed that USP35 is significantly overexpressed in various malignant tumors, including CRC (Figure 2A; Figure S2). Further clinical correlation analysis demonstrated a significant positive correlation between USP35 expression and both the tumor N stage and TP53 mutation status (Figure 2B, C), indicating a potential specific function in TP53‐mutated CRC. Validation using clinical samples, including immunohistochemical detection of 66 paraffin‐embedded CRC specimens and RT‐qPCR analysis of 39 fresh tissues, confirmed that USP35 expression is significantly higher in cancerous tissues compared to adjacent normal tissues (Figure 2D–F). Previous research has shown that USP35 inhibits ferroptosis in lung and kidney cancers and promotes cell proliferation and chemotherapy resistance in CRC [33, 34]. Our study further corroborated the proliferative effect of USP35 in CRC (Figure 2G). Analysis of the DepMap database also indicated that the proliferation of nearly all CRC cells was significantly inhibited following USP35 knockout (Figure 2H). These findings suggest that USP35 plays a crucial role in the pathogenesis and progression of CRC.

**FIGURE 3:**
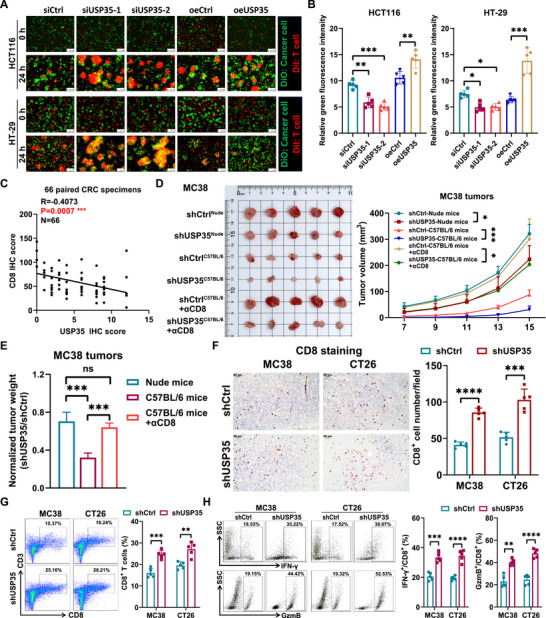
FIGURE 3 USP35 mediates suppression of T cell cytotoxicity against CRC cells. (A, B). Representative immunofluorescence results (A) and corresponding statistical analysis (B) show that USP35 inhibited T cell‐induced cytotoxicity on HCT116 and HT‐29 CRC cells. (C). Immunohistochemical analysis reveals a significant negative correlation between USP35 expression and infiltrating CD8^+^ T cells in human CRC specimens. (D). Tumor burden analysis of subcutaneously implanted USP35‐deficient MC38 mouse CRC cells in immunocompetent and immunodeficient mice. (E). MC38 tumor weights are normalized to the mean of corresponding control groups. n = 5. (F). Representative immunohistochemistry results and corresponding quantitative analysis display increased infiltration of CD8^+^ T cells in USP35‐deficient tumors compared to control tumors in a mouse model using MC38 and CT26 CRC cells. (G, H). Representative FACS analysis plots and quantification of the percentage of CD8^+^ T cells among CD45^+^ TILs (G) and IFN‐γ^+^, GZMB^+^ cells among CD8^+^ T cells (H) in MC38 and CT26 tumors. n = 5. si, siRNA; sh, shRNA; oe, overexpression; DiO, green fluorescent dye; DiI, red fluorescent dye. Data are presented as means ± SD. * *p* < 0.05, ** *p* < 0.01, *** *p* < 0.001, **** *p* < 0.0001, based on Student's *t* test (B, D–H) and Pearson *r* test (C).

Given the significant role of ID3 in promoting immune escape in CRC and considering USP35's function as a deubiquitinating enzyme of ID3, the role of USP35 in immune escape warrants further investigation. In vitro T cell killing assays demonstrated that CRC cells with USP35 knockdown exhibited increased sensitivity to CD8^+^ T cell‐mediated cytotoxicity (Figure 3A, B), accompanied by a significant increase in IFN‐γ and TNF‐α secretion (Figure S3A, B). Clinical specimen analysis revealed a significant association between high USP35 expression and reduced CD8^+^ T cell infiltration in the tumor microenvironment (Figure 3C; Figure S1D), as well as significant correlations with the expression levels of immune‐related factors such as TGF‐β, IFN‐γ, TNF‐α, and GzmB (Figure S3C). To validate these findings, we established USP35‐deficient MC38 and CT26 mouse models of CRC. The results demonstrated that USP35 knockdown significantly inhibited tumor growth in both immunodeficient nude mice and immunocompetent mice (Figure 3D; Figure S3D). Notably, in immunocompetent mice, the growth inhibitory effect of USP35‐deficient tumors was more pronounced (Figure 3E; Figure S3E), suggesting that the tumor‐promoting effect of USP35 is partially dependent on its regulation of the immune microenvironment. Further analysis revealed a significant increase in CD8^+^ T cell infiltration in the tumor tissues of the USP35 knockdown group (Figure 3F), along with a significantly higher proportion of IFN‐γ^+^CD8^+^ and GzmB^+^CD8^+^ cells in the tumor microenvironment, indicating CD8^+^ T cell activation and toxicity (Figure 3G, H). Concurrently, we observed lower intratumoral Treg frequencies and a increased M1/M2 macrophage ratio upon USP35 knockdown (Figure S3F–G). Collectively, these results indicate that USP35 not only promotes CRC cell proliferation by stabilizing the ID3 protein but also facilitates immune escape by modulating the tumor immune microenvironment, thereby playing a multifaceted role in the progression of CRC.

### 2.3 USP35 Exerts Its Regulatory Function on PD‐L1

**FIGURE 4:**
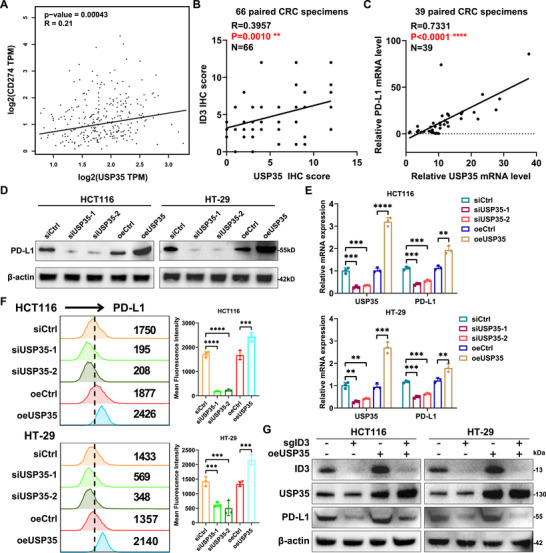
FIGURE 4 Tumor cell‐expressed USP35 enhances PD‐L1 expression. (A). The correlation between USP35 and PD‐L1 expression from the TCGA database is analyzed using the GEPIA platform. (B). Quantitative analysis depicts a significant positive correlation between the expressions of USP35 and PD‐L1 in paraffin‐embedded CRC specimens. n = 66. (C). RT‐qPCR analysis demonstrates a significant positive correlation between USP35 and PD‐L1 in human CRC specimens. n = 39. (D, E). Western blot (D) and RT‐qPCR (E) analysis illustrate the impact of USP35 expression on PD‐L1 levels in human CRC cells. (F). Flow cytometry analysis displays the influence of USP35 expression on cell membrane‐bound PD‐L1 in HCT116 and HT‐29 cells. (G). Western blot results show that the regulation of PD‐L1 expression by USP35 is significantly influenced by ID3 in CRC cells. si, siRNA; sg, sgRNA; oe, overexpression. Data are presented as means ± SD. ** *p* < 0.01, *** *p* < 0.001, **** *p* < 0.0001, based on Student's *t* test (E, F) and Pearson *r* test (A–C).

CRC cells have been documented to evade immune detection by overexpressing PD‐L1, which suppresses T cell function [14]. To explore the hypothesis that USP35 modulates CD8^+^ T cell activity by regulating PD‐L1 expression, an analysis of the TCGA database was undertaken. This analysis identified a significant positive correlation between USP35 and PD‐L1 expression in CRC tissues (Figure 4A). This correlation was further substantiated through IHC and RT‐qPCR analyzes in clinical CRC samples (Figure 4B, C; Figure S1D). To further understand this regulatory mechanism, a series of experiments were conducted. Initially, Western blot and RT‐qPCR analyzes were performed on human CRC cell lines, revealing that USP35 knockdown significantly decreased PD‐L1 expression, whereas USP35 overexpression increased PD‐L1 levels (Figure 4D, E). To determine PD‐L1's primary cell membrane localization and function, we further examined the effect of USP35 expression on cell membrane‐bound PD‐L1 in CRC cells using flow cytometry. Our results revealed that membrane PD‐L1 expression was positively correlated with changes in USP35 expression (Figure 4F). Additional investigations into USP35 overexpression in ID3‐knockout CRC cells showed that the regulatory effect of USP35 on PD‐L1 expression was notably reduced (Figure 4G), indicating that USP35 enhances PD‐L1 expression by stabilizing the ID3 protein.

### 2.4 USP35 Inhibits the Ubiquitination of ID3 by Targeting the K2/K30 Site

**FIGURE 5:**
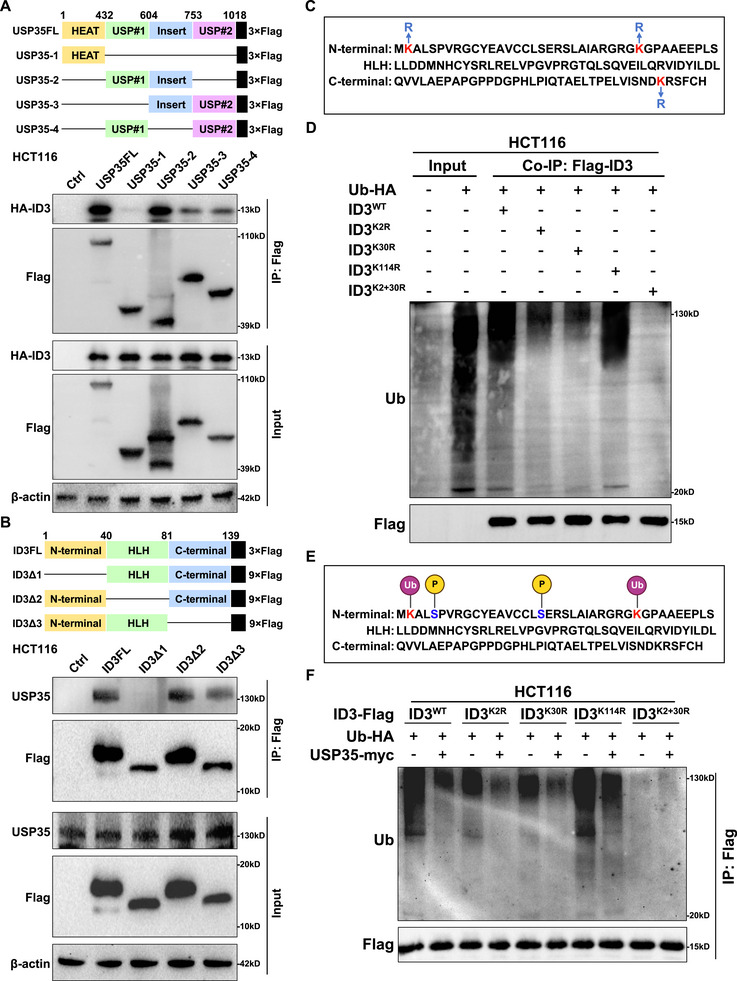
FIGURE 5 Mechanism by which USP35 inhibits ID3 ubiquitination. (A). Co‐IP using Flag‐tagged USP35 deletion mutant expression vector shows the interaction of ID3 with the USP#1 and the Insert domain of USP35 in HCT116 cells. (B). Co‐IP using the Flag‐tagged ID3 deletion mutant expression vector shows the interaction of USP35 with the N‐terminal domain of ID3 in HCT116 cells. (C). Schematic diagram of site‐directed mutagenesis of three lysine (K) residues in ID3 to arginine (R). (D). The effect of lysine (K) site‐directed mutagenesis to arginine (R) in ID3 on its ubiquitination level in HCT116 cells. (E). Schematic diagram of post‐translational modifications of ID3 protein. (F). USP35‐mediated deubiquitination of ID3 is affected by mutations at the K2 and K30 sites of ID3 in HCT116 cells.

To elucidate the molecular mechanism by which USP35 regulates the stability of the ID3 protein, a systematic investigation of the interaction patterns and key ubiquitination sites of the two proteins was conducted. Through Co‐IP experiments utilizing USP35 and ID3 mutants with various domain deletions [19, 35], it was determined that the USP#1 and Insert domains of USP35 exhibit specific binding affinity for the N‐terminal domain of ID3 (Figure 5A, B; Figure S4A, B). This finding suggests that the N‐terminal domain of ID3 may serve as the target site for USP35. Given that the specificity of the ubiquitin‐proteasome system relies on the covalent linkage of ubiquitin to the lysine ε‐amino group of target proteins, site‐directed mutagenesis was employed to convert the three lysine residues (K2, K30, and K114) in the ID3 amino acid sequence to arginine, and the levels of ID3 ubiquitination were subsequently assessed (Figure 5C). The results demonstrate that when the K2 and K30 sites of ID3 are individually mutated to arginine, the ID3 ubiquitination level is significantly reduced; when both are mutated together, ubiquitination is almost completely eliminated. The K114 mutation has no such effect (Figure 5D; Figure S4C), indicating that the K2 and K30 sites of ID3 are the primary sites for ID3 ubiquitination (Figure 5E). Further investigation into the deubiquitination sites of USP35 on ID3 revealed that mutation of the K114 site had no effect on USP35's regulation of ID3 ubiquitination. However, mutations at both the K2 and K30 sites impaired USP35's ability to regulate ID3 ubiquitination (Figure 5F; Figure S4D–F). The results of this study demonstrate that USP35 binds to the N‐terminus of ID3 via its USP#1 and Insert domains, specifically reducing ubiquitination at the K2/K30 sites to maintain ID3 protein stability. This finding provides a structural basis for understanding the role of the USP35‐ID3 regulatory axis in tumorigenesis.

### 2.5 IU1 Enhances Antitumor Immunity by Inhibiting the USP35‐ID3 Axis

**FIGURE 6:**
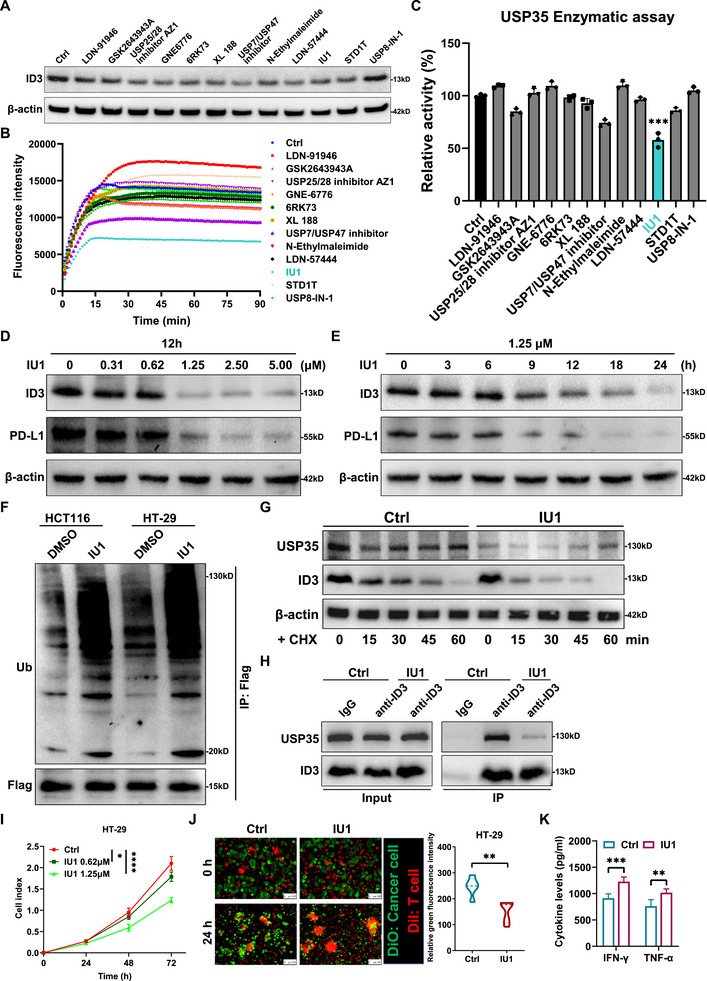
FIGURE 6 The deubiquitinating enzyme inhibitor IU1 enhances antitumor immunity by inhibiting the USP35‐ID3 axis. (A). Re‐verification of the effects of 12 inhibitors preliminarily screens from the deubiquitinating compound library on ID3 protein expression. (B). Alterations in fluorescence intensity resulting from the enzymatic hydrolysis of the Ub‐AMC fluorescent substrate following exposure to 12 inhibitors. (C). Quantification of inhibitor effects on USP35 enzyme activity by calculating the fluorescence intensity change rate during the linear phase of the in vitro DUB enzyme activity assay. (D, E). Western blot analysis of ID3 and PD‐L1 levels under different IU1 concentrations (D) and treatment durations (E). (F). Western blot analysis illustrates the impact of IU1 on ID3 ubiquitination levels in CRC cells. (G). The half‐life of ID3 protein following a 12‐hour treatment with 1.25 µM IU1 in HT‐29 cells. (H). Endogenous Co‐IP analysis reveals an attenuated USP35‐ID3 interaction in IU1‐treated HT‐29 cells. (I). CCK‐8 assay for assessing HT‐29 cell viability following IU1 treatment. (J). Representative immunofluorescence results and corresponding statistical analysis show that IU1 enhances T cell‐induced cytotoxicity on HT‐29 cells. (K). ELISA analysis of IFN‐γ and TNF‐α levels in the culture supernatant after IU1 treatment during the T cell‐induced cytotoxicity assay. DiO, green fluorescent dye; DiI, red fluorescent dye. Data are presented as means ± SD. * *p* < 0.05, ** *p* < 0.01, *** *p* < 0.001, **** *p* < 0.0001, based on Student's *t* test (B, D–H) and Pearson *r* test (C).

Given the established role of USP35 in facilitating tumor immune evasion through its deubiquitinating activity on ID3, a comprehensive screening approach was undertaken, utilizing a deubiquitinating enzyme inhibitor library comprising 56 compounds. This systematic investigation identified 12 compounds capable of inhibiting ID3 protein expression in HCT116 cells, including LDN‐91946, GSK2643943A, AZ1, GNE‐6776, 6RK73, XL 188, USP7/USP47 inhibitor, N‐Ethylmaleimide, LDN‐57444, IU1, STD1T, and USP8‐IN‐1 (Figure 6A; Figure S5, Table S1). A fluorescence substrate assay based on UB‐AMC was employed to evaluate the effects of these 12 inhibitors on USP35 enzyme activity. This assay demonstrated that IU1 significantly inhibited USP35 enzyme activity, suggesting its potential role in regulating ID3 protein stability by targeting USP35 (Figure 6B, C). Further studies demonstrated that IU1 suppressed ID3 expression in both dose‐ and time‐dependent manners: treatment with 1.25 µM significantly decreased ID3 protein levels, with effects initiating at 9 h and peaking after 24 h (Figure 6D, E). Notably, PD‐L1 expression was observed to be downregulated in response to the inhibition of ID3 by IU1 (Figure 6D, E). Concurrently, ID3 mRNA levels remained unchanged within 24 h post‐IU1 treatment (Figure S6A, B), suggesting that IU1 influences ID3 protein stability by modulating ubiquitination.

After establishing the optimal concentration and duration for IU1 treatment, it was discovered that the ubiquitination of the ID3 protein was markedly increased post‐treatment (Figure 6F), while its half‐life was significantly reduced (Figure 6G). Co‐IP experiments confirmed that IU1 treatment substantially weakened the interaction between USP35 and ID3 (Figure 6H). Following IU1 inhibition, overexpression of wild‐type USP35 restored CRC proliferation and rescued both ID3 and PD‐L1 levels, whereas the catalytically inactive mutant failed to do so, suggesting that the effects of IU1 are specifically mediated through USP35 (Figure S6C, D). Treatment with IU1 failed to modulate ID3 abundance in USP35 knockdown CRC cells (Figure S6E), demonstrating that the compound's effect on ID3 is strictly USP35‐dependent. Further functional assays revealed that IU1 not only suppresses the proliferation of CRC cells (Figure 6I), but also significantly boosts the cytotoxic activity of CD8^+^ T cells against these cancer cells (Figure 6J, K). These findings underscore the unique role of IU1 as a USP35 inhibitor and clarify its molecular mechanism in antitumor immunotherapy through the USP35‐ID3‐PD‐L1 axis.

### 2.6 IU1 Enhances the Antitumor Effect of PD‐L1 Antibody

**FIGURE 7:**
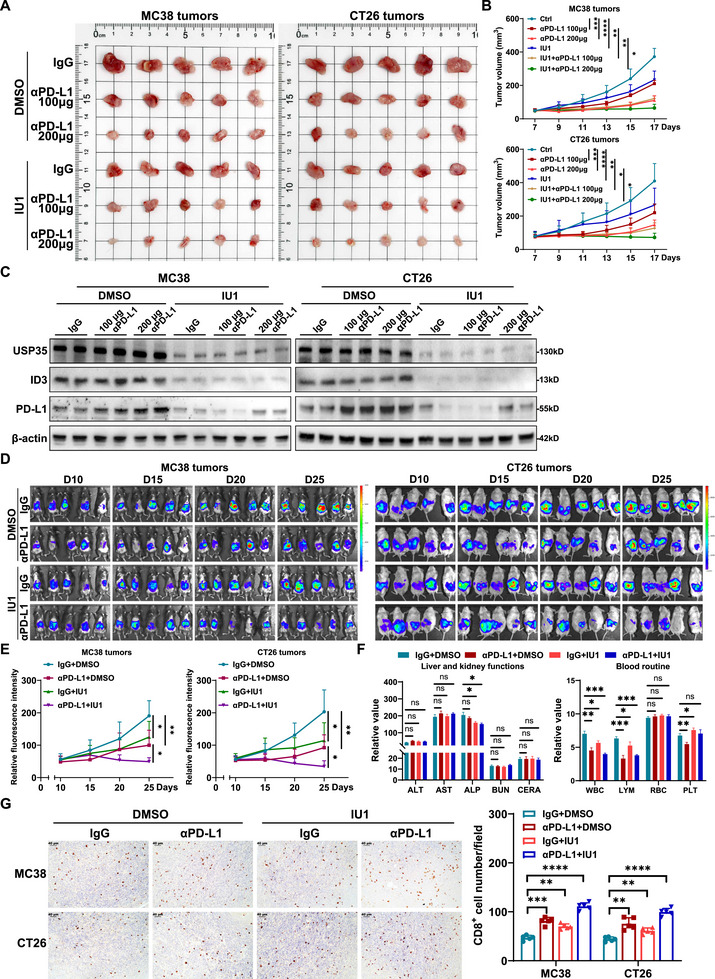
FIGURE 7 IU1 potentiates PD‐L1 antibody immunotherapy efficacy in vivo. (A). Tumor burden analysis in mice subcutaneously inoculated with MC38 and CT26 cells and treated with IU1 plus αPD‐L1. (B). Statistical analysis of tumor growth curves in tumor‐bearing mice shows that both IU1 and low‐dose (100 µg) or high‐dose (200 µg) αPD‐L1 therapy significantly inhibits the growth of MC38 and CT26 tumors, with the combination treatment exhibiting superior antitumor efficacy. (C). Western blot analysis of PD‐L1 expression in subcutaneously implanted MC38 and CT26 tumors following treatment. αPD‐L1 monotherapy significantly upregulates PD‐L1 expression, while IU1 suppresses the αPD‐L1‐induced upregulation of PD‐L1. (D). In vivo imaging depicting the growth of MC38 and CT26 tumors implanted in the cecum of mice. n = 5. (E). Statistical analysis of bioluminescence intensity curves from in vivo imaging demonstrates that IU1 enhances the therapeutic efficacy of αPD‐L1. n = 5. (F). Changes in liver and kidney function and blood routine in mice following treatment with IU1 and αPD‐L1. (G). IHC results and corresponding statistical analysis illustrate a significant increase in infiltrating CD8^+^ T cells within tumors upon treatment with IU1, αPD‐L1 monotherapy, and combination therapy. αPD‐L1, PD‐L1 monoclonal antibody; ALT, alanine aminotransferase; AST, aspartate aminotransferase; ALP, alkaline phosphatase; BUN, blood urea nitrogen; CERA, creatinine; WBC, white blood cell; LYM, lymphocyte; RBC, red blood cell; PLT, platelet. Data are presented as means ± SD. * *p* < 0.05, ** *p* < 0.01, *** *p* < 0.001, **** *p* < 0.0001, based on Student's *t* test.

To assess the in vivo inhibitory effect of IU1 on immune evasion in CRC, a subcutaneous tumor model was developed in immunocompetent mice treated with IU1, αPD‐L1 monotherapy, or a combination therapy. Since the effective concentration of IU1 on ID3 in vivo was unclear, we treated tumor‐bearing mice with escalating doses of IU1 and evaluated ID3 expression in excised MC38 tumors. Western blot analysis revealed that ID3 levels were significantly reduced when IU1 reached 100 µg per injection (Figure S7A),; therefore, we adopted this dose (100 µg IU1 every other day) for all subsequent animal studies. Under this regimen, both high‐dose and low‐dose αPD‐L1 monotherapy, as well as IU1 monotherapy, significantly curtailed tumor growth. However, the combination therapy group exhibited more pronounced effects, with IU1 significantly enhancing the tumor suppression rate of low‐dose αPD‐L1 (Figure 7A, B). Further analysis indicated that αPD‐L1 monotherapy feedback‐upregulates PD‐L1 expression in CRC, whereas IU1 combination therapy mitigates this adaptive resistance phenomenon (Figure 7C), suggesting that IU1 enhances the durability of PD‐L1 blockade therapy.

To further substantiate the clinical translational potential of IU1, an in situ tumor implantation model was developed in the cecum to replicate the immune microenvironment of CRC and assess the efficacy of combination therapy with IU1 and low‐dose αPD‐L1. The findings were consistent with the conclusions drawn from the subcutaneous tumor model, IU1 significantly augmented the antitumor effect of αPD‐L1 (Figure 7D, E). Post‐treatment analyzes of blood counts and liver and kidney function in mice revealed that IU1 had negligible effects on liver and kidney function but resulted in a decrease in ALP levels, the specific mechanism of which remains unclear. However, the combination therapy of IU1 and αPD‐L1 did not further exacerbate the decrease in ALP. While both IU1 and αPD‐L1 exhibited substantial inhibitory effects on lymphocytes, the combination therapy did not lead to an escalation of this toxicity. Moreover, while αPD‐L1 has been observed to cause a decrease in platelet (PLT) levels, IU1 has been shown to increase PLT levels (Figure 7F). In the analysis of the immune microenvironment of the in situ implanted tumors, it was found that CD8^+^ T cell infiltration was significantly higher in both the IU1 group and the αPD‐L1 group compared to the control group, with the highest CD8^+^ T cell infiltration observed in the combination therapy group (Figure 7G). Furthermore, ELISA and RT‐qPCR results demonstrated that the combination therapy group exhibited increased expression of CD8, IFN‐γ, TNF‐α, and GzmB, while TGF‐β expression was significantly reduced (Figure S7B–E). These results further confirm that IU1 can remodel the immune microenvironment of CRC, enhance tumor sensitivity to immunotherapy, and provide important experimental evidence for the clinical combination of IU1 and immune checkpoint inhibitors.

## 3 Discussion

As research continues to elucidate the role of ID3 in tumorigenesis and tumor progression, its function within tumors has become increasingly evident [19]. ID3 frequently acts as a pivotal molecular hub that facilitates tumor proliferation, sustains tumor stemness, and mediates drug resistance, among malignant phenotypes [25, 36, 37]. Tumor cells have been observed to adapt more effectively to the microenvironment within the body by overexpressing ID3, thereby promoting tumor progression [38]. However, current research on the regulatory mechanisms of ID3 remains significantly limited, particularly concerning its post‐translational modification regulatory network, which urgently requires further elucidation. This study systematically demonstrates that the ubiquitin‐proteasome pathway plays a predominant role in regulating ID3 protein homeostasis. In normal cells, ID3 may be rapidly ubiquitinated and degraded after fulfilling functions such as cell development; however, in tumor cells, due to dysfunction of the ubiquitin regulation system, ID3 cannot be normally degraded and accumulates abnormally, thereby driving malignant tumor progression. Therefore, elucidating the ubiquitin‐mediated regulatory mechanisms of ID3 is significant.

**FIGURE 8:**
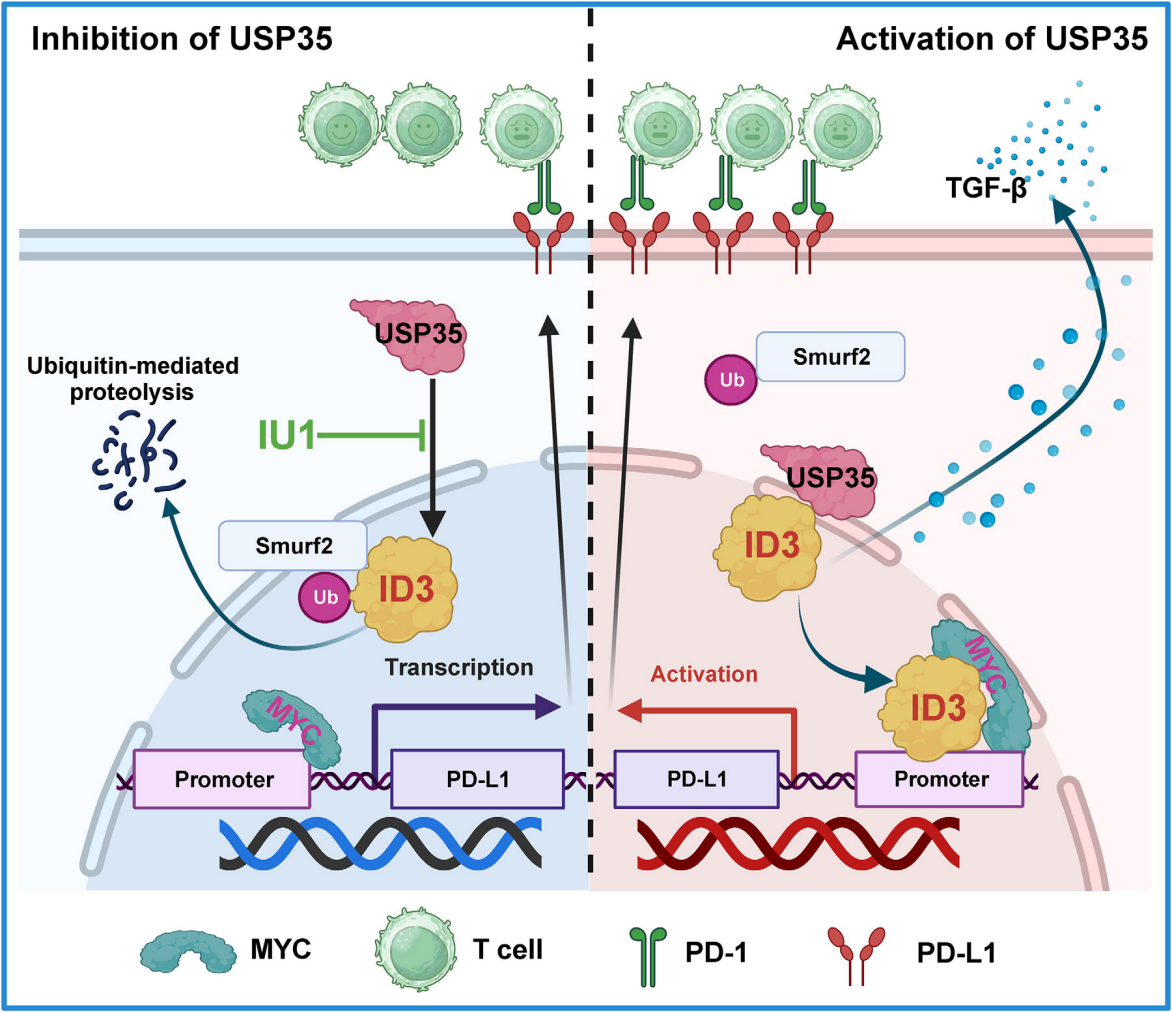
FIGURE 8 Schematic model illustrating how USP35 drives CRC immune escape by stabilizing ID3. USP35 stabilizes ID3 expression by deubiquitinating the K2/K30 site, thereby upregulating PD‐L1 and promoting immune escape in CRC. IU1, an inhibitor of USP35 enzyme activity, has been shown to inhibit USP35, thereby accelerating ID3 degradation, enhancing CD8^+^ T cell killing, and reversing the immunosuppressive microenvironment.

Previous research has suggested that Smurf2 may serve as the E3 ligase responsible for catalyzing ID3 ubiquitination [32]; however, investigations into the regulatory system governing ID3 deubiquitination have been notably lacking. In this study, a high‐throughput screening of 109 deubiquitinating enzymes was performed to evaluate their impact on ID3 expression, leading to the novel identification of USP35 as a key enzyme in the regulation of ID3 deubiquitination. As a prominent member of the deubiquitinating enzyme family, USP35 possesses the ability to form homodimers, with its catalytic activity dependent on this dimeric structure, and it maintains its own stability through an auto‐deubiquitination mechanism [39]. USP35 exhibits significant tissue specificity and pathological relevance. It has been shown to interact with Aurora B, regulating its stability via deubiquitination and thereby influencing cell mitotic processes [35]. Furthermore, USP35 is markedly overexpressed in various tumor tissues, including those associated with lung cancer [40], hepatocellular carcinoma [41], and breast cancer [42]. This overexpression is closely linked to reduced patient survival and increased tumor malignancy [43]. Functionally, USP35 primarily exerts its effects in tumors through its deubiquitinating enzyme activity. It has been demonstrated that this stabilization can promote lung cancer cell proliferation by stabilizing VEGFA and it regulates metabolic reprogramming in hepatocellular carcinoma by stabilizing PKM2 [44, 45]. Notably, USP35 enhances chemotherapy resistance by inhibiting ferroptosis in lung cancer [34], renal cell carcinoma [46], and breast cancer [33]. Additionally, studies have reported that USP35 can inhibit STING activity by deubiquitinating it, thereby attenuating the type I interferon response induced by chemotherapy drugs and promoting immune escape in ovarian cancer [47]. A significant finding in this study is the discovery that USP35 functions as a deubiquitinating enzyme for ID3, thereby stabilizing the ID3 protein and consequently enhancing PD‐L1 expression, thus facilitating immune evasion in CRC (Figure 8). This finding not only addresses a research gap in the deubiquitinating regulatory mechanism of ID3 but also provides a novel perspective for understanding the molecular basis of tumor immune escape.

In light of the pivotal function of the ubiquitin‐proteasome system in modulating ID3 expression, this study has further elucidates the principal ubiquitination sites of ID3, specifically the N‐terminal K2 and K30 lysine residues. This discovery significantly augments the post‐translational modification regulatory network of ID3, following the prior identification of phosphorylation sites. It is noteworthy that both K2 and K30 are situated within the N‐terminal domain of ID3, a finding that aligns with Co‐IP experimental results indicating that USP35 predominantly associates with the N‐terminal region of ID3. The identification of ID3 ubiquitination sites is crucial for understanding the biological implications of ID3 mutations. Mutations at these ubiquitination sites may hinder effective ubiquitination and degradation of ID3, potentially resulting in abnormal protein accumulation. Although ID3 mutation frequency is relatively low in CRC, it is significantly elevated in malignancies such as lymphoma [48]. Mutations at ubiquitination sites may directly enable ID3 to evade ubiquitin‐mediated degradation.

Numerous studies have corroborated the essential role of ID3 and its deubiquitinating enzyme USP35 in tumorigenesis and progression. However, the absence of specific small‐molecule inhibitors targeting this pathway significantly constrains related translational medical research. To address this limitation, the present study screened 56 deubiquitinating enzyme inhibitors and identified IU1 for the first time. This inhibitor facilitates ID3 ubiquitination and degradation by inhibiting USP35 enzyme activity, although IU1 also downregulates USP35 expression. Functional experiments revealed that IU1 exerts two primary effects; it significantly inhibits tumor cell proliferation in CRC models and enhances the cytotoxic effects of CD8^+^ T cells on tumor cells. Additionally, it augments the antitumor effects of immune checkpoint inhibitors. It is notable that USP35 plays a critical role in regulating ferroptosis, warranting further investigation into whether IU1 has the potential to induce ferroptosis and thereby synergize with chemotherapy drugs to produce antitumor effects. Furthermore, although IU1 is also an inhibitor of USP14, its IC50 for USP14 is 4–5 µM, and concentrations of 30–100 µM are typically required in cellular assays to effectively inhibit USP14 activity [49, 50]. We found that treatment with 10.0 µM of IU1 for 12 h did not alter USP14 and its substrate SLC7A11 levels [51] (Figure S8). In contrast, our findings indicate that IU1 can significantly inhibit USP35 activity at a concentration of 1.25 µM following 9 h of exposure. This clear dose‐response relationship demonstrates that IU1 exhibits substantially higher target specificity for USP35 inhibition compared to USP14. In light of these findings, the development of targeted intervention strategies for the ID3 ubiquitination site and the formulation of more efficient combination therapy regimens are identified as significant future research directions. These findings provide a robust foundation for translational research on the IU1‐USP35‐ID3‐PD‐L1 signaling axis, as well as potential targets and a theoretical basis for the development of novel anticancer drugs.

## 4 Conclusions

In summary, this study uncovers a novel USP35‐ID3‐PD‐L1 regulatory axis that plays a critical role in promoting immune evasion in CRC. USP35 functions as a deubiquitinating enzyme that stabilizes ID3 by removing ubiquitin modifications at the K2 and K30 sites, thereby preventing its degradation. Stabilized ID3 enhances PD‐L1 expression, leading to suppressed CD8⁺ T cell activity and a more immunosuppressive tumor microenvironment. Importantly, we identified IU1 as a potent inhibitor of USP35, which promotes ID3 degradation, reduces PD‐L1 levels, and enhances the efficacy of anti‐PD‐L1 immunotherapy. These findings not only deepen our understanding of post‐translational regulation of ID3 but also provide a promising therapeutic strategy for improving immunotherapy responses in CRC patients.

## 5 Experimental Section

### 5.1 Ethics Statement

The clinical tissue samples utilized for the purpose of verification in this study were collected in accordance with the principles outlined in the Declaration of Helsinki, and approval for this purpose was received from the Ethics Committee of Fujian Cancer Hospital (Ethics No. K2023‐107‐01). The laboratory mice employed in the experiment were supplied by the Experimental Animal Center of Fujian Provincial Hospital (License No. SYXY(Min)2023‐0005) in accordance with established animal welfare guidelines and were handled by certified laboratory personnel (Fujian Association for Laboratory Animal Science No. 2023‐0028). Their usage was sanctioned by the Experimental Animal Ethics Committee of Fujian Provincial Hospital.

### 5.2 Cell Culture

The human CRC cell lines, HCT116 (RRID:CVCL_0291) and HT‐29 (RRID:CVCL_0320), as well as the mouse CRC cell lines, MC38 (RRID:CVCL_B288) and CT26 (RRID:CVCL_7254), were purchased from the Cell Bank of the Type Culture Collection Committee of the Chinese Academy of Sciences (Shanghai, China). All cells were maintained in 1640 or DMEM culture medium (Hyclone) supplemented with 10% fetal calf serum (Gibco) and incubated in a 37°C, 5% CO_2_ incubator. All cell lines were subjected to kit‐based testing (Lonza) in order to confirm their freedom from mycoplasma contamination.

### 5.3 Plasmids, siRNA and CRISPR/Cas9 Genome Editing

Utilizing conventional molecular biology techniques, cDNA was employed as a template to construct expression plasmids for ubiquitin, ID3, and USP35 with HA or Flag tags. The expression plasmid vector employed in this study was pcDNA3.1, which was transferred into cells using Liposome 3000. The shRNA targeting mouse USP35 and the sgRNA targeting human ID3 were designed by Hanbio Biotechnology (Shanghai) Co., Ltd. The shRNA targeting human USP35 was purchased from MCE Inc. (#HY‐RS15546). The sgRNA was then cloned into the LentiCRISPR v2 vector. The specific sequences of these vectors were as follows: mm‐shUSP35, 5′‐CGCTACCTCATCCTCACACTA‐3′; hs‐sgID3, 5′‐CGCCTGCGGGAACTGGTACC‐3′. The corresponding empty vector was used as a control. The expression of the antibiotic transferase from the mouse shUSP35 plasmid results in puromycin resistance of cells, and the assessment of this resistance was used for the purpose of screening. The efficacy of the knockdown or overexpression of the target gene was confirmed by Western blot analysis.

### 5.4 Immunohistochemistry (IHC)

The tissue specimens utilized in the immunohistochemical study were obtained from Fujian Cancer Hospital between 2020 and 2023. The study comprised a total of 66 patients with a clinical and pathological diagnosis of CRC who had not previously received treatment. The specimens were processed using an immunohistochemistry detection kit (MXB, #KIT‐9701), and the paraffin sections of the tissue were baked at 60°C in a water‐free electric constant‐temperature incubator. Following dewaxing and rehydration, high‐temperature and high‐pressure antigen retrieval was performed using citric acid antigen retrieval solution. The sections were then treated with 50 µl of Reagent A (a peroxidase inhibitor), followed by incubation and washing. Subsequently, 50 µl of Reagent B (normal non‐immune animal serum) was added, followed by an additional round of incubation and washing. The sections were then treated with USP35 (Invitrogen #PA537232), ID3 (Invitrogen #PA576793), PD‐L1 (Proteintech #66248‐1), or CD8 (Affinity #AF5126) antibodies overnight at 4°C. Subsequently, 50 µl of secondary antibody and 50 µl of Reagent D were added. The section were then washed, after which 50 µl of DAB reagent was added for color development. The results were subsequently interpreted independently by two pathologists. The immune response score for tumor cells was determined by quantifying the product of the positive cell proportion score (0‐4) and the staining intensity score (0‐3).

### 5.5 Real‐Time Quantitative PCR (RT‐qPCR)

Total RNA was isolated using the standard Trizol extraction method. Compared to cells, tissues must undergo repeated grinding in a mortar with liquid nitrogen prior to RNA extraction, followed by lysis with Trizol. Following the quantitative extraction of total RNA, 1 µg of RNA was reverse transcribed into cDNA using a reverse transcription kit for subsequent quantitative PCR analysis. The relative abundance of mRNA was calculated using the comparative threshold cycle method (2^−∆∆Ct^), with β‐actin as the internal control for normalization. The sequences of primers were listed in Table S2 of the Supporting Information.

### 5.6 Western Blot

After trypsin digestion and centrifugation, CRC cells were collected and lysed using a cell lysis buffer. Tissue samples from humans and mice were ground in liquid nitrogen, sonicated, and then centrifuged for collection. Protein quantification was performed using the BCA method. Equal amounts of protein were loaded onto SDS‐PAGE gels and then transferred to PVDF membranes (Millipore). The membranes were then subjected to immunoblotting using blocking BSA, primary antibodies, and secondary antibodies. Protein blots were analyzed using a chemiluminescence imaging system (Bio Rad). The primary antibodies used were as follows: USP35 (Invitrogen #PA5 44966), ID3 (Calbioreagent #M100), PD‐L1 (Sigma #SAB4301882), Ubiquitin (CST #3933), Flag‐tag (Sigma #F9291), HA‐tag (Sigma #H9658), β‐actin (CST #8457), USP14 (CST #11931T), SLC7A11 (Proteintech #26864‐1‐AP).

### 5.7 Protein Half‐Life Detection Assay

CRC cells were transfected with empty vector (oeCtrl), wild‐type USP35 plasmid (oeUSP35) or catalytically inactive USP35 mutant (oeΔUSP35) for 48 h, grown to 80% confluence, and then treated with 100 µg ml^−1^ cycloheximide (CHX, MCE #HY‐12320) to block de‐novo protein synthesis. After adding CHX, cell samples were collected at 0, 15, 30, and 45 min, followed by lysis and protein extraction for quantitative analysis. Western blot analysis was then used to detect the levels of the ID3 protein. By comparing the expression levels of the ID3 protein at different time points, the time corresponding to a reduction in its concentration to half the initial value was determined, which represents the half‐life of the ID3 protein.

### 5.8 CCK8 Proliferation Assay

Cells were seeded at a density of 5 × 10^3^ cells per well in 96‐well plates and incubate at 37°C in a 5% CO_2_ incubator for 24 h until the cells adhere. Different concentrations of drugs or treatment factors were added according to the experimental design and incubation was continued for 24–72 h. After incubation, 10 µL of CCK‐8 reagent was directly added to each well, gently mixed by shaking, and incubated in the dark for 1–4 h. The OD of light at 45 nm was measured using a microplate reader. A well containing culture medium without cells as the blank control was the baseline. The relative cell survival rate = experimental group OD value/control group OD value × 100%. A set up six replicate wells per group was used to reduce experimental error.

### 5.9 Isolation and Culture of CD8^+^ T Cells

Ficoll lymphocyte separation solution was added to a centrifuge tube then peripheral blood diluted with PBS was slowly mixed in. After centrifugation, the middle layer composed of peripheral blood mononuclear cells (PBMC) was retrieved. Once the PBMC were resuspended in PBS, CD8^+^ microbeads were introduced to facilitate the sorting of CD8^+^ T cells using the Miltenyi MACS system. The sorted CD8^+^ T cells were cultured in KBM 581 medium (Corning) supplemented with CD3/CD28 monoclonal antibodies and IL‐2 (Kingsley Pharmaceutical, China) for activation and expansion in a CO_2_ incubator at 37°C for a duration of seven days.

### 5.10 T Cell‐Induced Cytotoxicity Assay

We seeded 1×10⁵ CRC cells into 12‐well plates. After the cells adhered, DiO cell membrane green fluorescent dye (Beyotime, C1038) was added. After staining, 4 × 10⁵ CD8⁺ T cells pre‐stained with DiI live cell red fluorescent dye (Beyotime, C1036) were added. Set the co‐culture ratio of CRC cells to T cells at 1:4 and incubate at 37°C in 5% CO_2_ for 24 h. After co‐culture, the intensity of red fluorescence (marking live T cells) and green fluorescence (marking CRC cells) was detected using a fluorescence microscope, and the relative intensity of green fluorescence was used to quantify the level of CRC cell death. The co‐culture medium was then collected, centrifuged at 1000×g for five minutes to separate the supernatant, and the concentrations of cytokines such as IFN‐γ and TNF‐α secreted by T cells in the supernatant were detected using the ELISA method to further evaluate the cytotoxic effect of CD8⁺ T cells on CRC cells and their immune activation status.

### 5.11 ELISA

After the T cell cytotoxicity assay the co‐culture supernatant was collected and centrifuged at 1500 rpm for 5 min. The supernatant was frozen at ‐80°C and stored for future analysis after dilution 5‐fold with dilution buffer. Following the instructions in the ELISA kit manual (Sino Biological, China), 100 µl of standard samples at gradient concentrations were added to each well of an 8‐well standard plate. Supernatant samples, 100 µl, were added to the designated wells and the plates incubated at room temperature for two hours. Then 200 µl per well of freshly prepared substrate solution was added and plates incubated at room temperature in the dark for 20 min. Stop solution was then added, mixed thoroughly, and the OD value read at 450 nm. The cytokine concentration of the test samples was determined from the standard curve.

### 5.12 Flow Cytometry

Harvest CRC cells, centrifuge at 1500 rpm for five minutes, and remove the supernatant. Resuspend the cells in 100 µl PBS, then add 10 µl CD274 PE antibody (BD #557924). PE isotype antibody was used as a control. Incubate the cells at room temperature for 20 min. After incubation, centrifuge at 1500 rpm for five minutes, remove the supernatant, and wash twice with 100 µl PBS. Determine the proportion of PE‐positive cells by flow cytometry. Analyze the data using FlowJo software.

### 5.13 Immune Cell Isolation and Infiltrated CD8^+^ T Cell Activity Analysis

Tumor tissues were minced in RPMI 1640 basal medium and enzymatically digested with collagenase buffer (containing 0.5 mg ml^−1^ type IV collagenase and 1 mg ml^−1^ DNase I) at 37°C for 30 min. The digestion process was terminated by adding an equal volume of RPMI 1640 medium containing 10% fetal bovine serum (FBS). Subsequently, the digested tissue was filtered through a 70 µm cell strainer to obtain a single‐cell suspension. Tissue‐infiltrating lymphocytes were separated using Ficoll density gradient centrifugation. Non‐viable cells were excluded from analysis using the Zombie UV Fixable Viability Kit (Biolegend #423108). For intracellular cytokine staining, cells were stimulated for six hours with conditioned medium containing the Leukocyte Activation Cocktail with GolgiPlug (BD #550583) prior to antibody staining. After surface staining with the relevant antibodies, cells were fixed and permeabilized for 30 min using Fix and Permeabilize Solution (BD #555028). Samples were analyzed using a BD Fortessa X‐20 flow cytometer, and data were processed using FlowJo software. The fluorescently conjugated antibodies used in this study were as follows: FITC CD3 (BioLegend #100306), Percp/Cy5.5 CD4 (Involtrogen #45‐0042‐82), APC/Cy7 CD8 (Involtrogen #47‐0081‐82), APC IFNγ (BioLegend #505810), EF450 Granzyme B (Involtrogen #48‐8898‐82).

The infiltrating Treg and M1/M2 macrophages, mononuclear cells isolated by Ficoll gradient separation were first surface‐stained with CD4 (BioLegend #100405) and CD25 (BioLegend #102011). After fixation and permeabilization (BioLegend #424401), intracellular Foxp3 (BioLegend #126403) staining was performed to identify Treg cells. Macrophage subsets were gated on CD68 (BioLegend #137007), followed by CD86 (BioLegend #159203) for M1 and CD206 (BioLegend #141703) for M2 classification.

### 5.14 Co‐Immunoprecipitation (Co‐IP)

Harvested CRC cells were transfected with the target plasmid, cells were lysed, and incubated with labeled antibodies at 4°C overnight. Next, the antigen‐antibody complex was combined with washed protein A/G agarose at room temperature for one hour. The complex was washed twice with washing buffer, each time for five minutes, and finally washed with purified water. The supernatant was discarded, 100 µl of elution buffer added to the centrifuge tube, and incubated at room temperature for 10 min. Then add 10 µl of neutralization solution. The supernatant obtained after centrifugation was the immunoprecipitation mixture. Western blot analysis was used to detect protein interactions in the mixture.

### 5.15 Deubiquitinating Enzyme Activity Assay

HCT116 cells transfected with the 3×Flag‐USP35 plasmid were treated with a deubiquitinating enzyme inhibitor and resuspended in suspension buffer. The cell suspension was lysed using an ultrasonic disruptor, and the lysate was incubated with Flag magnetic beads at 4°C for four hours. The magnetic beads were washed with suspension buffer, and five volumes of 3×Flag peptide working solution were added at 4°C and inverted to mix. Competitive elution was performed for one hour, and the eluted supernatant was collected for protein quantification. In vitro DUB activity assays were performed at room temperature using the lysis‐sensitive fluorescent substrate Ub‐AMC (Boston Biochem, U‐550). All reactions were performed in 96‐well plates. Fluorescence intensity was measured at an excitation wavelength of 355 nm and an emission wavelength of 426 nm using a Spectramax M3 microplate reader (Molecular Devices). The initial substrate stock solution was prepared according to the manufacturer's instructions and diluted to a working solution concentration of 5 µM in assay buffer (20 mM HEPES pH 7.5, 150 mM NaCl, 1 mM DTT). The enzyme solution was diluted to a concentration of 6 µM in assay buffer. The reaction system was prepared using 50 µl of assay buffer, 20 µl of substrate working solution, and 30 µl of enzyme solution. Measurements were taken once per minute for 90 min, followed by measurements every 1.5 min. Enzyme activity was determined from the linear phase of the time‐absorbance curve (constant ΔA between points) by calculating ΔA min^−1^, then multiplying by the theoretical K value (U L^−1^). Relative activity was expressed as (experimental/control × 100%). Data from three measurements were plotted and analyzed using GraphPad software.

### 5.16 Subcutaneous Tumor Implantation Model In Vivo

Six‐ to eight‐week‐old female mice were randomly divided into six groups, with five mice in each group. During the logarithmic growth phase, CRC cells were collected from the control group and the USP35 knockdown group. Subsequently, each mouse was subcutaneously injected with 2 × 10⁶ cells. To deplete CD8^+^ T cells, mice were administered 200 µg of anti‐mouse CD8α (Bio X Cell, #BE0061) or IgG2b (Bio X Cell, #BE0090) via intraperitoneal injection two days prior to tumor cell injection. This regimen was maintained every four days until the end of the experiment. To evaluate the efficacy of the inhibitors, PD‐L1 (Bio X Cell #BE0101) was administered intraperitoneally at a dose of 100 or 200 µg every two days, and IU1 (MCE, HY‐13817) was administered intraperitoneally at a dose of 100 µg every two days, starting one week after tumor cell injection. Tumor size was measured every two days starting from day seven (tumor volume = length × width × width/2). On day 17, mice were euthanized, tumor tissue was extracted, total RNA was isolated for RT‐qPCR analysis, and proteins were extracted for Western blot analysis to assess the expression levels of target proteins.

### 5.17 Cecal Tumor Implantation Model In Vivo

Female mice aged 6–8 weeks were used. The mice were randomly divided into six groups, with five mice in each group. All surgeries were performed in a biosafety cabinet equipped with disinfected instruments to ensure sterility. Mice were anesthetized via intraperitoneal injection of a 0.3% sodium pentobarbital solution. The limbs of the mice were secured to a sterile surgical table with adhesive tape, and eye ointment was applied to prevent retinal damage. The surgical site was disinfected after hair removal. An incision was made in the abdominal cavity using forceps and scissors to expose the cecum. Using an insulin syringe, a suspension of 2×10⁶ mouse CRC cells transfected with luciferase was slowly injected into the subserosal layer of the cecum. After suturing with sterile sutures, dexamethasone was administered to the mouse to reduce inflammation caused by the surgery. Use a heating pad to warm the mouse and allow it to regain consciousness. Starting from day 10 post‐tumor cell inoculation, PD‐L1 (Bio X Cell #BE0101) was administered intraperitoneally at a dose of 100 µg every two days, and IU1 (MCE #HY‐13817) was administered intraperitoneally at a dose of 100 µg every two days, with isotype IgG as the control. In vivo bioluminescence imaging was performed on the mice every five days. At the end of the experiment, the mice were euthanized, and tumor tissue was collected for weight measurement and subsequent analysis.

### 5.18 In Vivo Bioluminescence Imaging

Using the PE IVIS Lumina X5 small animal in vivo imaging system, the growth status of cancer cells in mice was evaluated using bioluminescence imaging technology. Prior to imaging, each mouse anesthetized with isoflurane gas was administered an intraperitoneal injection of 100 µl of D‐luciferin potassium salt bioluminescence substrate (25 mg ml^−1^), followed by imaging 10 min later. Pseudocolor images of active luciferase in each mouse were acquired using the following parameters: pixel width and height 1, fractal factor 2, luminescence exposure 10 s, f‐value 4. Signal intensity was quantified using Living Image software.

### 5.19 Bioinformatics Analysis

This study employs multiple bioinformatics platforms to conduct multidimensional data analysis. The UALCAN online platform (https://ualcan.path.uab.edu/) based on the TCGA database was used to systematically analyze the expression profiles of USP35 in CRC and other tumors. As an interactive cancer genomics data analysis tool, this platform enables precise comparisons of gene expression differences between tumor tissues and normal tissues. To analyze the impact of USP35 on proliferation in CRC cells, genomic background data and small molecule perturbation sensitivity information for tumor cell lines were obtained from the DepMap platform (https://depmap.org/portal/home/#/). Using the GEPIA platform (http://gepia.cancer‐pku.cn/), we specifically analyzed the gene expression correlation between USP35 and PD‐L1. This platform was specifically designed for cancer gene expression profiling association analysis and supports extended functions such as Pearson/Spearman correlation tests and survival analysis.

### 5.20 Statistical Analysis

All statistical analyzes were conducted using GraphPad Prism 8.0. A p‐value of less than 0.05 was considered to be significant. Each experiment was replicated thrice. In order to assess the statistical significance between two groups of samples, a two‐tailed Student's t‐test was utilized. For the purpose of comparing samples from the same patient, a paired t‐test was employed. The correlation between the two detection indicators within the same group of samples was examined using correlation analysis and linear regression, with the aim of fitting a straight line. In instances where data normality could not be assured, either paired or unpaired Wilcoxon tests were applied. The presentation of all group data was as means, with error bars representing standard errors of the mean (SEM). Statistical analysis and graphical representation were performed using GraphPad Prism software.

### 5.21 Ethics Approval and Consent to Participate

The clinical tissue samples utilized for the purpose of verification in this study were acquired in accordance with the principles outlined in the Declaration of Helsinki, and approval for this purpose was received from the Ethics Committee of Fujian Cancer Hospital (Ethics No. K2023‐107‐01). The mice employed in the experimental setting were provided and cared for by the Experimental Animal Center of Fujian Provincial Hospital in accordance with established animal welfare guidelines. Their usage was sanctioned by the Experimental Animal Ethics Committee of Fujian Provincial Hospital.

## Author Contributions

W.C., L.W., and H.F. contributed equally to this work. C.H. wrote the manuscript, prepared figures, and conducted statistical analysis. C.H. and Y.Y. contributed to the study concept and experimental design. W.C., L.W., and H.F. performed experiments and conducted statistical analysis. L.L., R.L., F.L., and L.Z. coordinated the study. F.W. conducted mouse experiments.

## Conflicts of Interest

The authors declare no conflicts of interest.

## Supporting information




**Supporting File 1**: advs73675‐sup‐0001‐SuppMat.pdf.

## Data Availability

The data that support the findings of this study are available in the supplementary material of this article.
