# Supplementary material for: USP35 Acts as a Deubiquitinating Enzyme for ID3 to Promote Immune Escape in Colorectal Cancer
Source: Adv Sci (Weinh). 2026 Jan 4;13(16):e16588. doi: 10.1002/advs.202516588 (PMC13042420; doi:10.1002/advs.202516588)
Supplement: Supplementary file 1 — Supporting File 1: advs73675‐sup‐0001‐SuppMat.pdf. [file ADVS-13-e16588-s001.pdf]

Table S1 Quantitative analysis of deubiquitinating enzyme inhibitors on ID3 expression

No.	Inhibitors' name	Relative protein level
0	Ctrl	1.00±0.13
1	HBX41108	2.84±0.22
2	USP7-IN-13	0.96±0.11
3	**LDN-91946**	**0.73±0.09***
4	HBX19818	1.02±0.14
5	**GSK2643943A**	**0.71±0.08***
6	**USP25/28 inhibitor AZ1**	**0.66±0.12***
7	USP28-IN-4	1.12±0.21
8	LCAHA	2.46±0.34
9	BC-1471	0.96±0.17
10	CMPD-39	1.21±0.18
11	IU1-47	0.91±0.14
12	ML-323	1.06±0.21
13	P005091	0.96±0.09
14	**GNE-6776**	**0.53±0.11****
15	**6RK73**	**0.59±0.16****
16	USP7-IN-9	0.81±0.32
17	**XL 188**	**0.47±0.21****
18	EOAI3402143	0.89±0.18
19	**USP7/USP47 inhibitor**	**0.49±0.08*****
20	6-Thioguanine	0.80±0.26
21	OTUB2-IN1	0.82±0.18
22	KSQ-4279	0.89±0.21
23	XL177A	0.93±0.19
24	USP14-IN-1	1.04±0.11
25	ML364	3.18±0.32
26	Degrasyn	2.18±0.21
27	**N-Ethylmaleimide**	**0.62±0.13****

No.	Inhibitors' name	Relative protein level
28	**LDN-57444**	**0.64±0.07******
29	BAY-805	1.24±0.12
30	IU1-248	1.32±0.15
31	8RK64	1.29±0.16
32	USP7-IN-1	1.18±0.21
33	NSC632839	4.16±0.36
34	USP5-IN-1	1.22±0.24
35	GRL0617	3.29±0.27
36	**IU1**	**0.14±0.05********
37	FT206	0.98±0.13
38	GNE-6640	0.97±0.16
39	USP30 inhibitor 11	1.02±0.18
40	TCID	0.99±0.23
41	PR-619	0.98±0.11
42	USP8-IN-2	0.97±0.09
43	USP7-IN-8	1.07±0.14
44	JAMM protein inhibitor 2	1.01±0.15
45	BAY 11-7082	0.97±0.14
46	Vialinin	0.95±0.10
47	Spautin-1	1.04±0.21
48	P 22077	0.91±0.18
49	MF-094	0.94±0.19
50	Capzimin	1.02±0.09
51	**STD1T**	**0.52±0.11*******
52	USP-IN-3	1.21±0.25
53	FT3967385	0.98±0.09
54	**USP8-IN-1**	**0.68±0.24****
55	RA-9	1.12±0.16
56	STAMBP-IN-1	0.85±0.28

* $P < 0.05$, ** $P < 0.01$, *** $P < 0.001$, **** $P < 0.0001$, based on Student's *t* test.

Table S2 The sequences of primers for RT-qPCR

Gene name	Forward sequence	Reverse sequence
Human USP35	CGTCACCTCCTTCTTCCCTAA	CTCCTTCTCCTGCTCCTGTAG
Human ID3	CAGCGCGTCATCGACTACA	CGTTGGAGATGACAAGTTCCG
Human PD-L1	AGGCCGAAGTCATCTGGACA	TGTTGATTCTCAGTGTGCTGGT
Human TGF-β	CACTCCCGTGGCTTCTAGTG	CTGGCGAGCCTTAGTTTGGA
Human IFN-γ	AGGTCATTCAGATGTAGCGGATA	TTCCTTGATGGTCTCCACACT
Human GzmB	TGCGAATCTGACTTACGCCAT	TGAGACTTTGGTGCAGGCTC
Human CD8	TACCTCTCCCAAAACAAGCC	AGTAGCCCTCGTTCTCTCGG
Human TNF-α	CACCACCATCAAGGACTCAAAT	TCAGGGAAGAATCTGGAAAGGT
Human β-actin	TGACGTGGACATCCGCAAAG	CTGGAAGGTGGACAGCGAGG
Mouse ID3	CTGCTACGAGGCGGTGTG	CGTCCAAGAGGCTAAGAGGC
Mouse PD-L1	ATCCTGTTGTTCCTCATTGTAGTG	CGTCTCCTCGAATTGTGTATCATT
Mouse TGF-β	CACTCCCGTGGCTTCTAGTG	CTGGCGAGCCTTAGTTTGGA
Mouse IFN-γ	AAGACAATCAGGCCATCAGC	CTGGACCTGTGGGTTGTTGA
Mouse GzmB	TGTGCTATGTGGCTGGTTGG	AAGCACGTTTGGTCTTTGGG
Mouse CD8	GGATTGGACTTCGCCTGTGATA	TGTGGTAGCAGATGAGAGTGATG
Mouse TNF-α	GCCGATGGGTTGTACCTTGT	TCTTGACGGCAGAGAGGAGG
Mouse β-actin	TGTCCACCTTCCAGCAGATGT	AGCTCAGTAACAGTCCGCCTAG

Figure S1

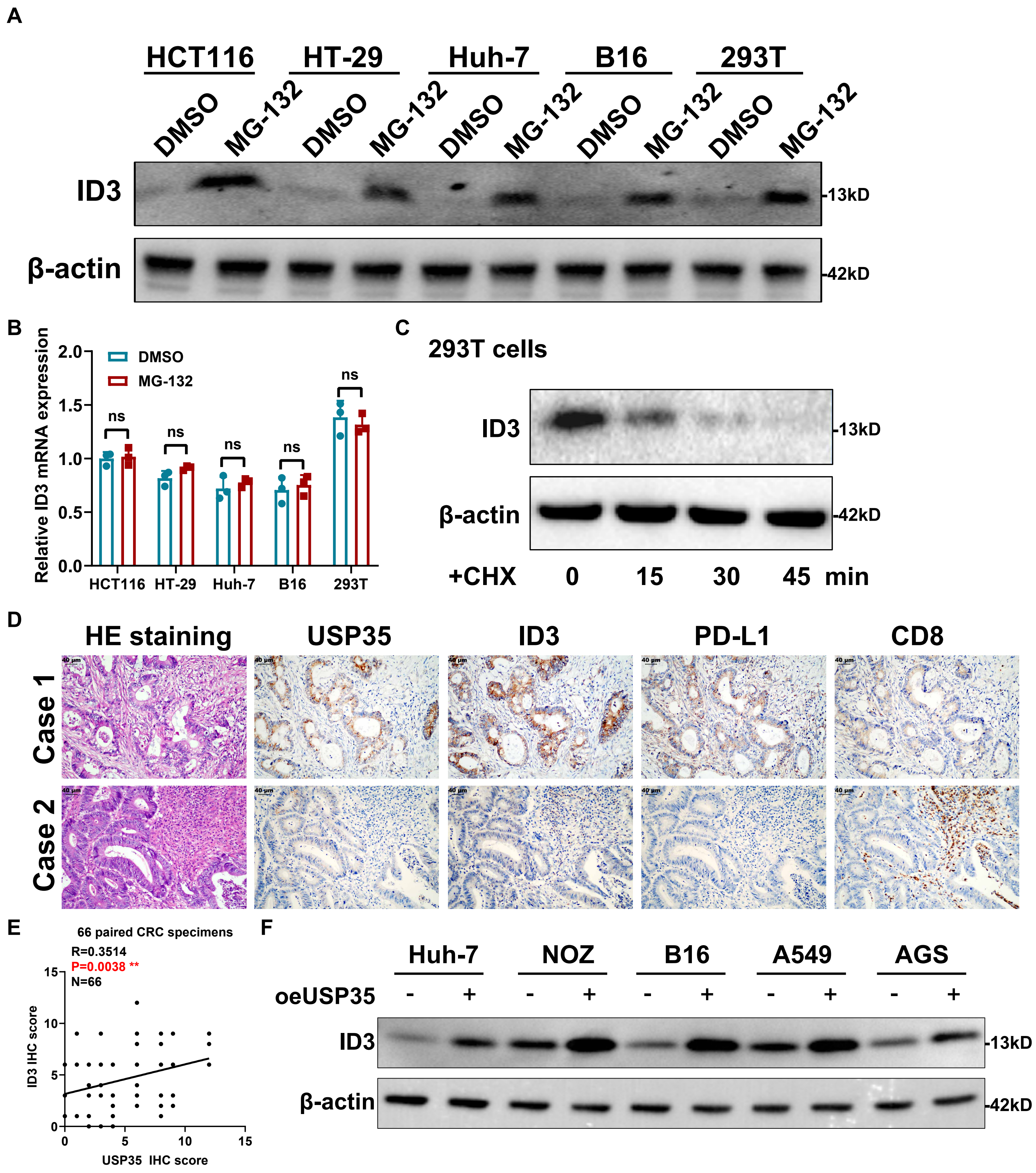

Figure S1. USP35 inhibits the ubiquitination and degradation of ID3. A. Western blot analysis illustrating the impact of MG-132 on ID3 levels in cancer cells. **B.** RT-qPCR analysis illustrating the impact of MG-132 on ID3 mRNA levels in human cancer cells. **C.** The half-life of ID3 protein. **D, E.** Representative immunohistochemistry results (**D**) and corresponding quantitative analysis (**E**) depict a significant positive correlation between the expressions of USP35 and ID3 in paraffin-embedded CRC specimens. n=66. **F.** Western blot analysis illustrating the impact of USP35 on ID3 levels in cancer cells. Huh-7, hepatocellular carcinoma cell; NOZ, gallbladder cancer cell; B16, melanoma cell; A539, lung adenocarcinoma cell; AGS, gastric carcinoma cell; oe, overexpression. ** $P < 0.01$, based on Pearson r test.

Figure S2

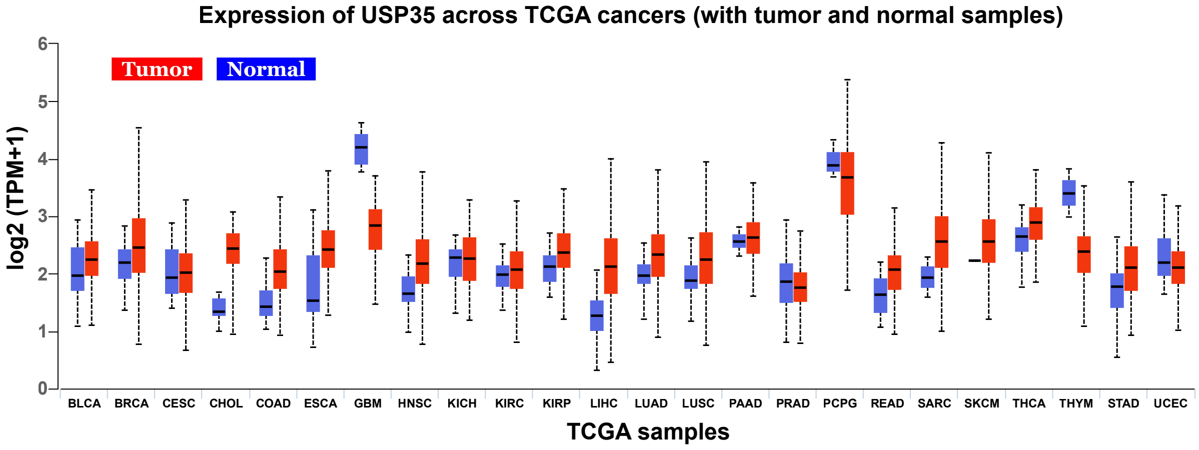

Figure S2. Pan-cance view of USP35 expression across TCGA database.

Figure S3

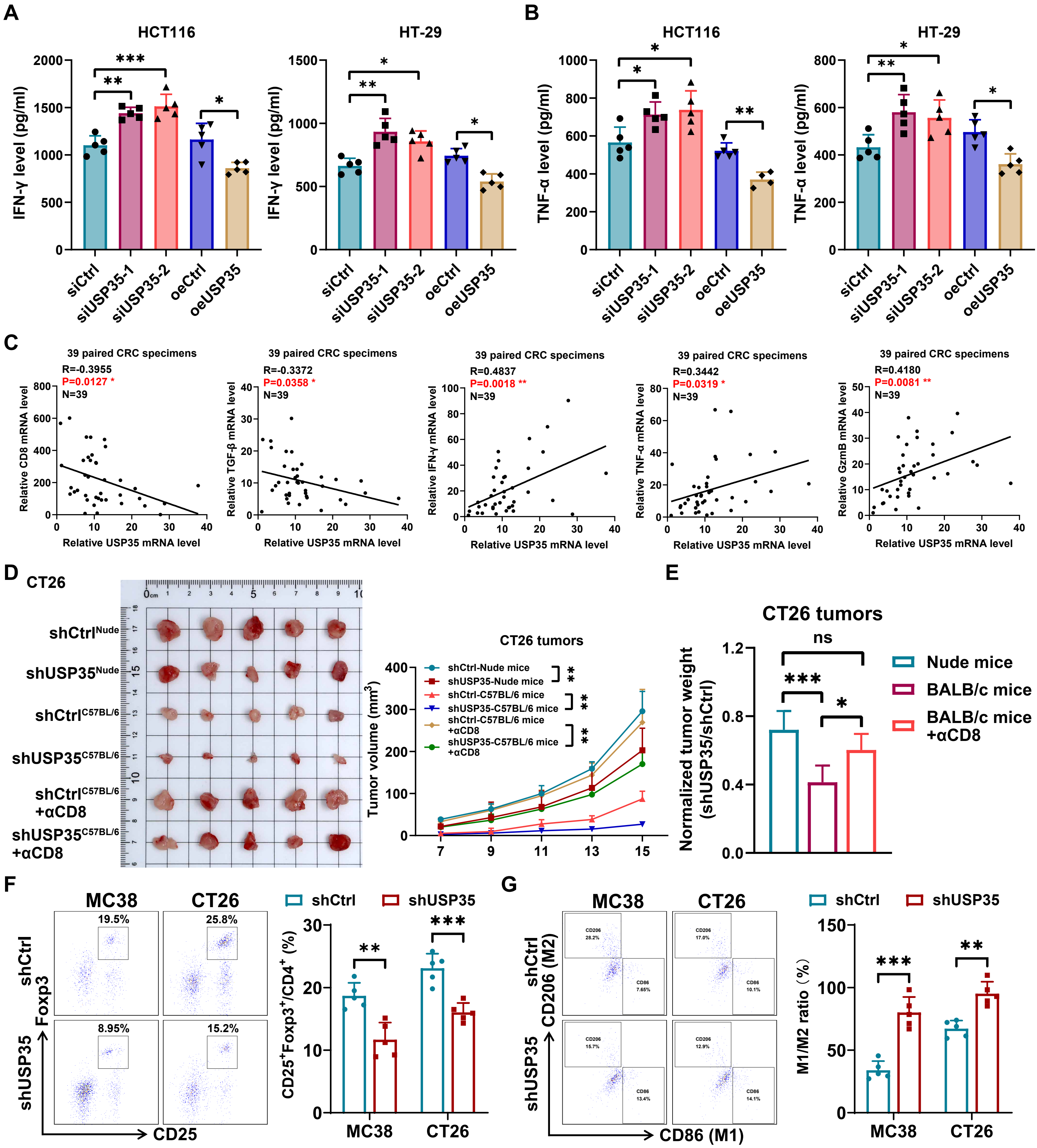

Figure S3. USP35 plays a promoting role in CRC immune evasion. A, B. ELISA analysis of IFN-γ levels (**A**) and TNF-α levels (**B**) in the culture supernatant during the T cell-induced cytotoxicity assay. **C.** Statistical analysis demonstrating a significant correlation between the expressions of USP35 mRNA and CD8, TGF-β, IFN-γ, TNF-α, GzmB mRNA in human CRC tissue samples. n=39. **D.** Tumor burden analysis of subcutaneously implantes USP35-deficient CT26 mouse CRC cells in immunocompetent and immunodeficient mice. **E.** CT26 tumor weights are normalized to the mean of corresponding control groups. **F, G.** Representative FACS analysis plots and quantification of the percentage of Treg (gating on CD4) (**F**) and M1/M2 macrophage ratio (gating on CD68) (**G**) in MC38 and CT26 tumors. n=5 per group. * $P < 0.05$, ** $P < 0.01$, *** $P < 0.001$, based on Pearson r test (C) and Student's t test (A-B, D-G).

Figure S4

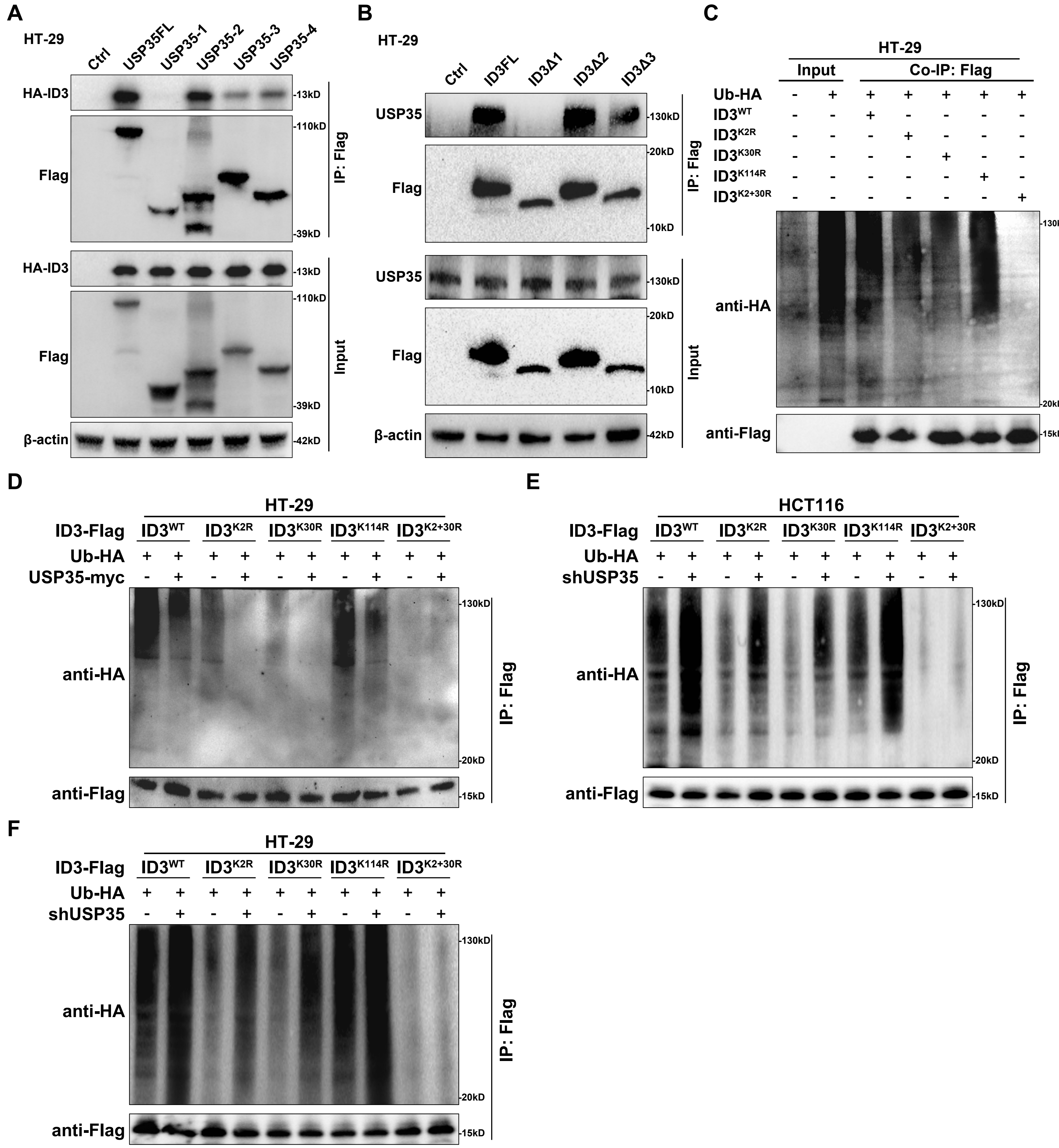

Figure S4. Determination of ID3 ubiquitination sites by USP35 in HT-29 cells. A. Co-IP using Flag-tagged USP35 deletion mutant expression vector shows interaction of ID3 with the USP#1 and Insert domain of USP35 in HT-29 cells. **B.** Co-IP using Flag-tagged ID3 deletion mutant expression vector shows interaction of USP35 with the N-terminal domain of ID3 in HT-29 cells. **C.** The effect of lysine (K) point mutation to arginine (R) in ID3 on its ubiquitination level. **D.** USP35 over-expression alters ID3 ubiquitination at K2 and K30 sites. **E, F.** USP35 knockdown alters ID3 ubiquitination at K2 and K30 sites in the HCT116 (**E**) and HT-29 (**F**) cells.

Figure S5

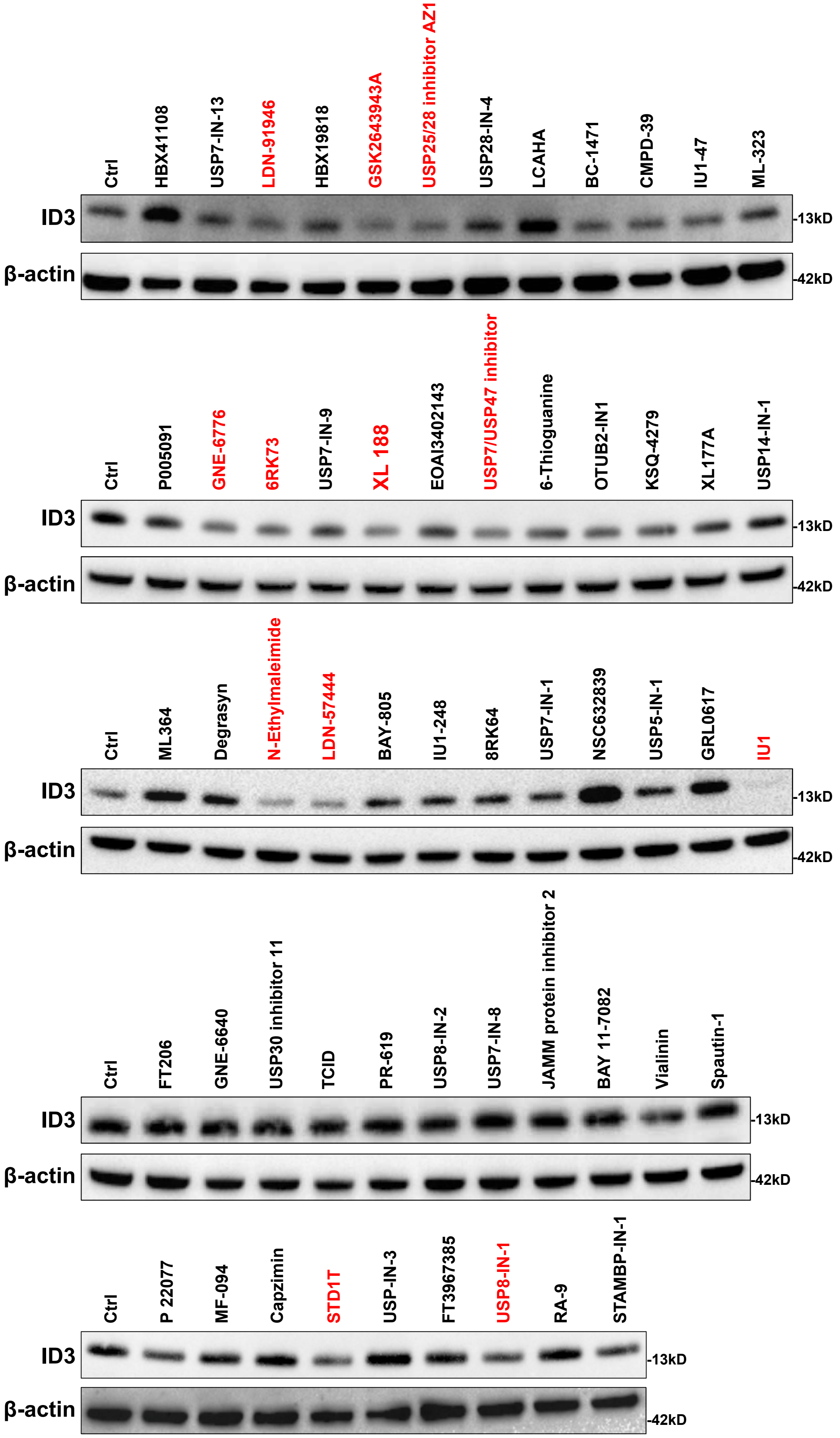

Figure S5. Screening the effects of 56 deubiquitinating enzyme inhibitors in the deubiquitinating compound library on ID3 expression.

Figure S6

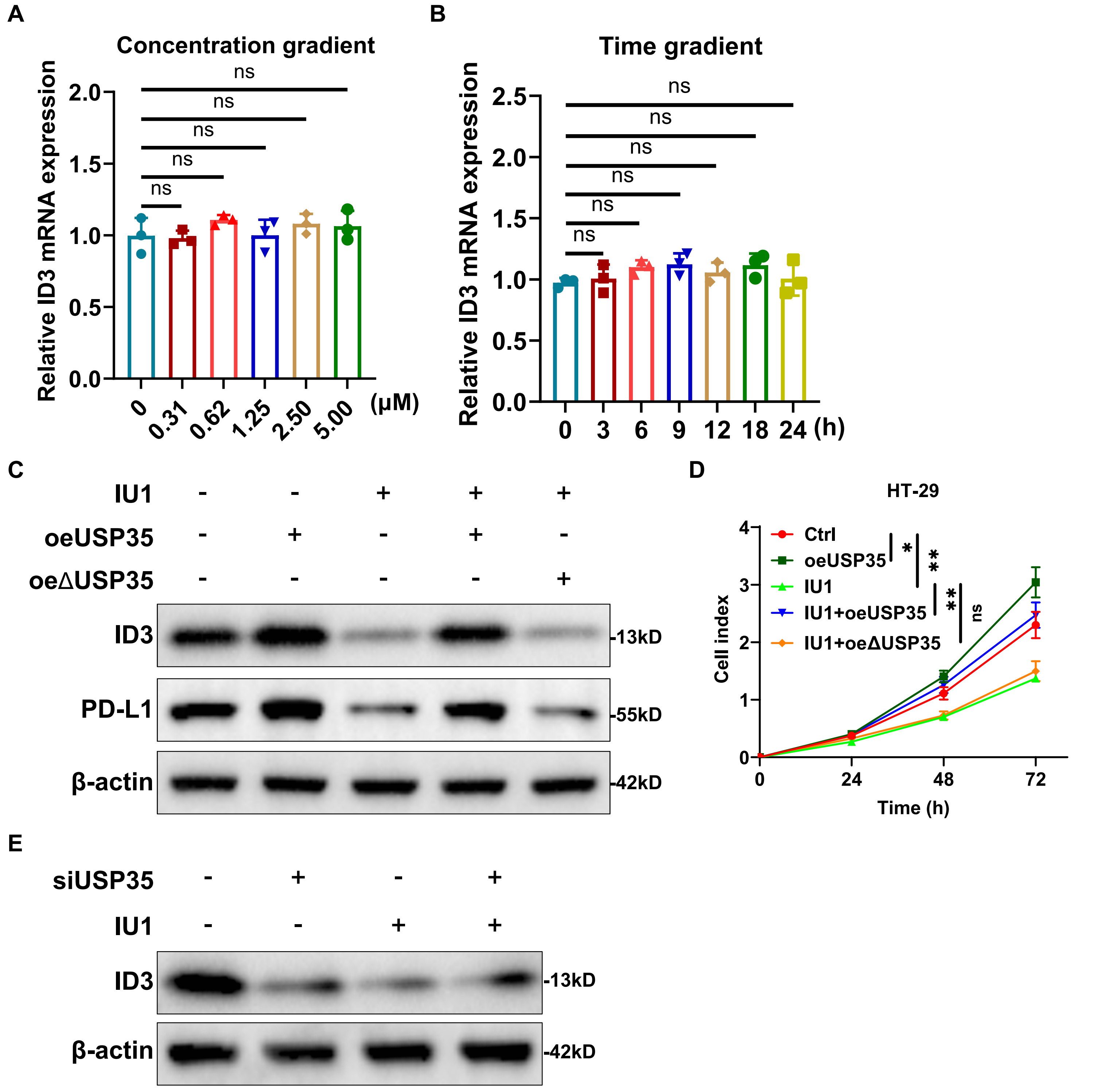

Figure S6. IU1 Regulates ID3 and PD-L1 Expression *via* USP35. A. RT-qPCR detection shows that different concentrations of IU1 had no effect on ID3 mRNA expression. n=5. **B.** RT-qPCR detection shows that IU1 had no effect on ID3 mRNA expression at different time points. n=5. **C.** Western blot analysis of ID3 and PD-L1 levels following IU1 treatment and overexpression of USP35 or its catalytically inactive mutant. **D.** CCK-8 assay for assessing HT-29 cell viability following IU1 treatment and upon overexpression of wild-type or catalytically inactive USP35. **E.** Western blot analysis of ID3 levels in USP35 knockdown HT-29 cells treated with IU1. oe, overexpression; ΔUSP35, catalytically inactive USP35. * $P < 0.05$, ** $P < 0.01$, based on Student's *t* test.

Figure S7

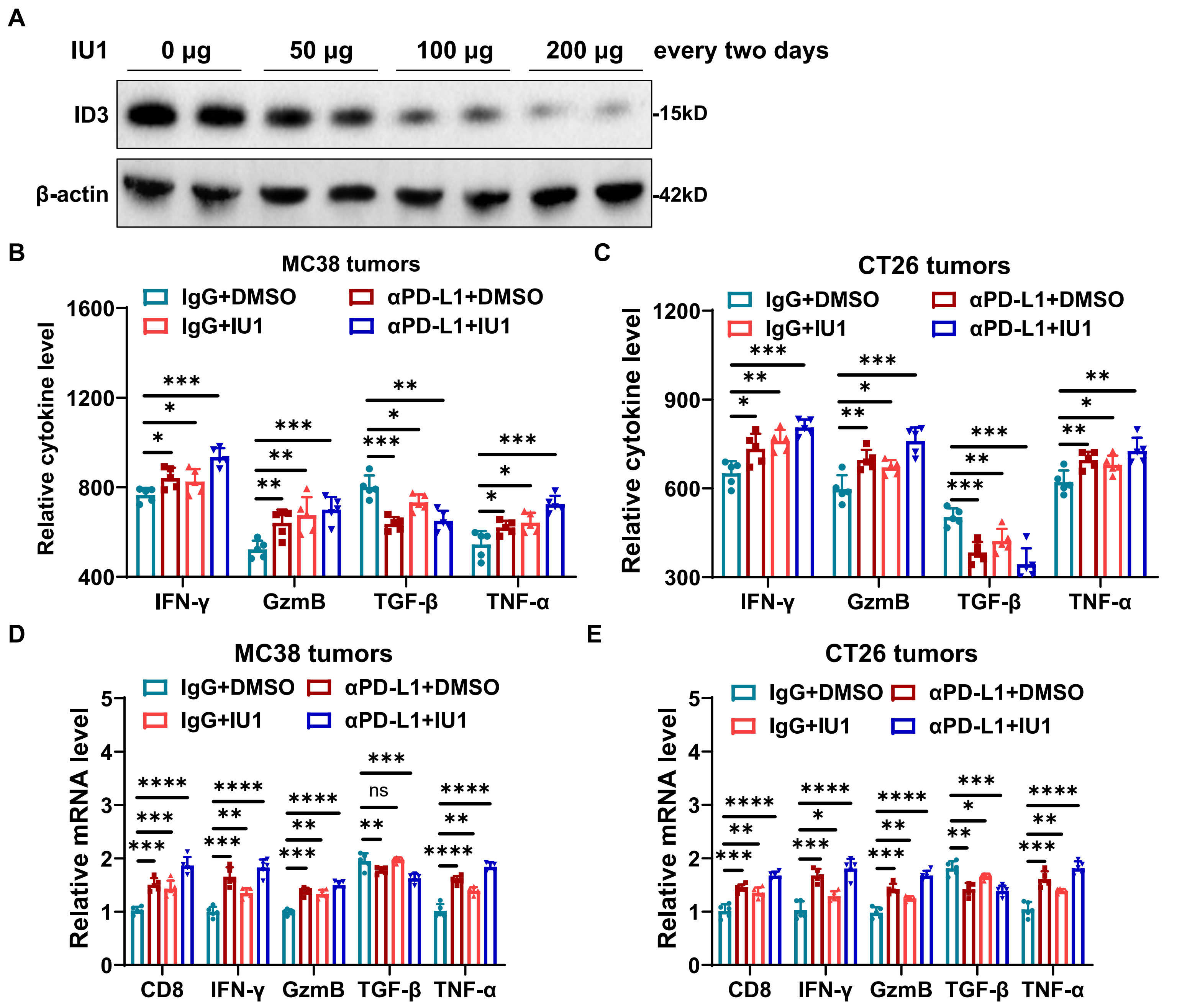

Figure S7. USP35 plays a promoting role in CRC immune evasion. A. Western blot analysis of ID3 levels in MC38 tumors from mice treated with varying concentrations of IU1. **B, C.** ELISA analysis of IFN-γ, GzmB, TGF-β, and TNF-α levels in the MC38 (**B**) and CT26 (**C**) tumors after treatment. **D, E.** The mRNA levels of CD8, IFN-γ, GzmB, TGF-β, and TNF-α in the MC38 (**D**) and CT26 (**E**) tumors after treatment. n=5 per group. * $P < 0.05$, ** $P < 0.01$, *** $P < 0.001$, **** $P < 0.0001$, based on Student's *t* test.

Figure S8

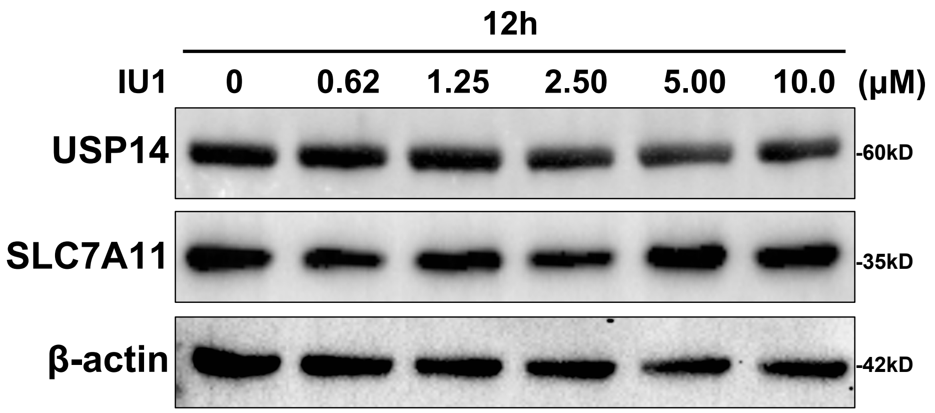

Figure S8. Low-dose IU1 does not affect USP14. Western blot analysis of USP14 and its substrate SLC7A11 levels following IU1 treatment in HT-29 cells.
